# Development of *o*-aminobenzamide salt derivatives for improving water solubility and anti-undifferentiated gastric cancer

**DOI:** 10.3389/fphar.2023.1118397

**Published:** 2023-07-11

**Authors:** Shuang Li, Yanli He, Xuelin Li, Yongxia Xiong, Yan Peng, Chengkun Wang, Linsheng Zhuo, Weifan Jiang, Xianzhou Lu, Zhen Wang

**Affiliations:** ^1^ School of Pharmaceutical Science, Hengyang Medical School, University of South China, Hengyang, Hunan, China; ^2^ Department of Pain Rehabilitation, The Affiliated Nanhua Hospital, Hengyang Medical School, University of South China, Hengyang, Hunan, China; ^3^ Department of Hepatobiliary Surgery, The Affiliated Nanhua Hospital, Hengyang Medical School, University of South China, Hengyang, Hunan, China; ^4^ Hunan Province Key Laboratory of Tumor Cellular & Molecular Pathology, Cancer Research Institute, Hengyang Medical School, University of, South China; ^5^ Postdoctoral Station for Basic Medicine, Hengyang Medical School, University of South China, Hengyang, Hunan, China

**Keywords:** *o*-aminobenzamide derivate, salt formation, undifferentiated gastric cancer, antitumor, water solubility

## Abstract

**Background:** Gastric cancer is one of the cancers with wide incidence, difficult treatment and high mortality in the world, especially in Asia and Africa. In our previous work, a novel *o*-aminobenzamide analogue **F8** was identified as an early preclinical candidate for treatment of undifferentiated gastric cancer (IC_50_ of 0.26 μM for HGC-27). However, the poor water solubility of compound **F8** prevents its further progress in preclinical studies.

**Aim:** To improve the water solubility and drug-likeness of **F8** via salt formation.

**Method:** Different acids and **F8** were reacted to obtain different salt forms. Physicochemical property screening, pharmacokinetic property research, and antitumor biological activity evaluation *in vitro* and* in vivo* were used to obtain the optimal salt form with the best druggability.

**Results:** our continuous efforts have finally confirmed **F8·2HCl** as the optimal salt form with maintained *in vitro* antitumor activity, improved water solubility and pharmacokinetic properties. Importantly, the **F8·2HCl** displayed superior* in vivo* antitumor efficacy (TGI of 70.1% in 75 mg/kg) in HGC-27 xenograft model. The further immunohistochemical analysis revealed that **F8·2HCl** exerts an antitumor effect through the regulation of cell cycle-related protein (CDK2 and p21), apoptosis-related protein Cleaved Caspase-3, proliferation marker Ki67, and cell adhesion molecule E-cadherin. In addition, **F8·2HCl** showed acceptable safety in the* in vivo* acute toxicity assay.

**Conclusion:** Salting is an effective means to improve the drug-like properties of compound **F8**, and **F8·2HCl** can serve as a promising therapeutic agent against undifferentiated gastric cancer.

## 1 Introduction

Gastric Cancer (GC) is one of the most common cancers in the world, although morbidity and mortality rates have declined in the United States and Europe in recent decades, it remains a serious disease in Asia and Africa. And its mortality rate ranks third among malignant tumors (Ajani et al., 2022). Its 5-year survival rate is around 20%, posing a serious threat to human health (Fang et al., 2021; Guo et al., 2021). In 2018, the number of newly diagnosed cases of stomach cancer is well over one million, and the number of deaths is increasing every year (Zhao et al., 2016; Zhang and Yu, 2020). There are many types of gastric cancer because of different degrees of differentiation, including moderately differentiated and highly differentiated gastric cancers. The lower the degree of differentiation, the higher the degree of malignancy in poorly differentiated (Lu et al., 2021; Singh et al., 2021). Due to the high degree of malignancy, undifferentiated gastric cancer is prone to local invasion and distant metastasis, with strong invasiveness, high recurrence rate and poor prognosis, and high risk of lymph node metastasis (Lee and Chung, 2020; Yang et al., 2022). Therefore, the therapeutic effect of undifferentiated gastric cancer is very poor, and there are few drugs that can be used for clinical treatment.

In recent decades, more and more drugs are being approved for the clinical treatment of gastric cancer (Lei et al., 2022). The commonly used anti-gastric cancer drugs are cytotoxic drugs, such as Tegafur, Doxifluridine, and Capecitabine (prodrugs of 5-Fluorouracil), etc., while they have many shortcomings such as lack of selectivity, serious adverse reactions, carcinogenic and teratogenic (Sharma, 2007; Okines et al., 2009). In addition, Trastuzumab is also used as a first-line treatment for stomach cancer, but has a serious side effect that can lead to heart failure (Hyun Cheol Chung et al., 2020; Janjigian et al., 2020). At present, the second- and third-line chemotherapy drugs clinically used in the treatment of gastric cancer mainly include perbrolizumab, ramucirumab and apatinib. Although these drugs have obvious efficacy, they all have relatively serious side effects, such as cardiac insufficiency, hematotoxicity and certain damage of liver and kidney function (Smyth et al., 2020). Therefore, it is imperative to study new anti-gastric cancer drugs with low toxicity, high efficiency and strong selectivity, especially for anti-undifferentiated gastric cancer (Shiotsuki et al., 2022).

**FIGURE 1:**
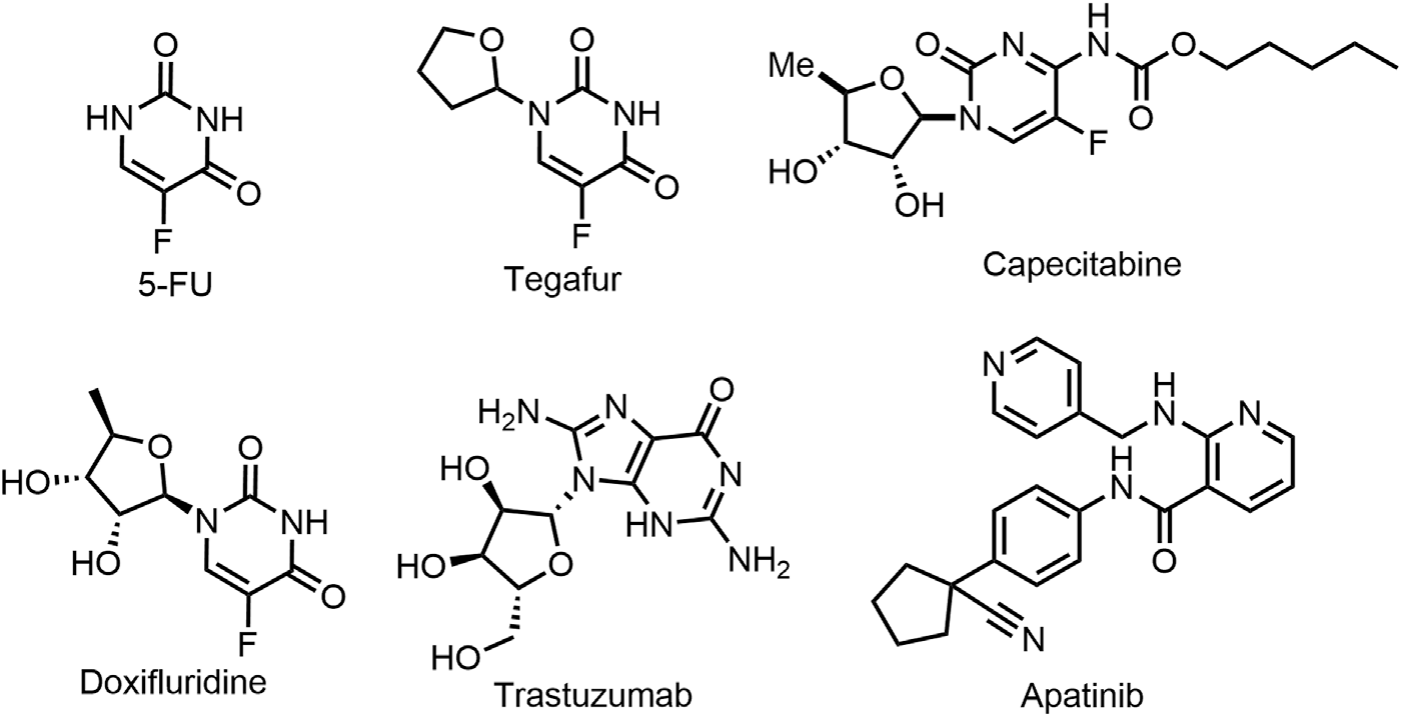
Chemical Structures of representative chemotherapeutics in clinical treatment of gastric cancer.

In previous work, we have developed a series of novel *o*-aminobenzamide derivatives, which exhibited potent anticancer effects against a variety of gastric cancer cells (Feng et al., 2022; Lu et al., 2022). Among them, compound **F8** has been proved to have the strongest anti-undifferentiated gastric cancer activity (IC50 = 0.26 μM), especially inhibiting the cell cycle and inducing apoptosis of human undifferentiated gastric cancer HGC-27 cells. Moreover, compound **F8** showed significantly better anti-xenograft tumor activity than capecitabine *in vivo*, with less toxicity. Preliminary studies have shown that the cyclin-dependent kinase 16 (CDK16) is a potential target of compound **F8**, which could upregulate the downstream effector p27 of CDK16, regulate cell cycle- and apoptosis-related proteins, and further exert the anticancer activity. Although compound **F8** has a significant inhibitory effect on transplanted tumor, it also has some defects due to poor water solubility, such as poor bioavailability, low exposure level and short half-life, which hinder the medicinal properties and clinical development of this compound. Aiming at the problem of low water solubility, salt formation is the key method to solve these problems (Figure 1).

Nearly half of all drug molecules are delivered in the form of salt (Acharya et al., 2018). Traditionally, improving solubility is one of the basic reasons for adopting salt forms, but numerous studies have shown that a unique salt form may have an impact far beyond solubility. It can improve some drugs that do not ideal physical chemical or biological pharmaceutical properties, such as solubility, dissolution or change of drugs to reduce moisture absorption and improve stability, change the melting point, and improve the grinding performance, easy preparation, purification and improve permeability, achieve sustained or targeted drug delivery, and so on (Gupta et al., 2018). Each salt form of a drug has unique properties. The final determination of salt form is to find a balance between physical and chemical properties and biological and pharmaceutical properties. Therefore, the selection of drug salt type starts from the selection of counter ions, and then the selection of crystallization conditions to prepare the corresponding salt type, which has an important guiding effect on the selection of solid dosage form with appropriate pharmaceutical properties. Salt formation of active pharmaceutical ingredients (APIs) is an integral part of the formulation development process, and salt selection requires a well-designed screening strategy that meets the basic and desirable criteria for formulating salt screening criteria (Bastin et al., 2000; Elder et al., 2013). Selecting the ideal salt type can improve the solid-state properties of the API and reduce the time consuming and expensive burden of formulation development. The counter ions of the salt used can have a positive effect on the suitability of the drug in various dosage forms by improving the properties of the preparation (Elder et al., 2013; Gupta et al., 2018). Selecting an optimal salt form for development is a critical step in ensuring the efficient and successful development of a robust product.

Therefore, in view of the poor water solubility of compound **F8**, this work synthesized compound **F8** into salt, because this is the most feasible way to improve water solubility. We further conducted a comprehensive antitumor activity study *in vitro* and *in vivo* on **F8·2HCl**, confirmed that the water solubility of **F8·2HCl** was more than 50 times higher than that of compound **F8**, and the pharmacokinetic properties *in vivo* were also improved. The exposure level of **F8·2HCl** was higher than that of compound **F8**, the half-life and Tmax were significantly increased, and the bioavailability was also significantly improved. Most importantly, the anti-xenograft tumor activity of **F8·2HCl** was much higher than that of compound **F8**. Therefore, the salt formation of compound **F8** is the key means to improve its efficacy.

## 2 Materials and methods

### 2.1 Chemicals

All the chemicals used in the experiment are commercially available, and all chemical reactions follow the synthetic route. Reactions were carried out under argon unless otherwise stated. NMR spectroscopy was performed using a Bruker 500 M NMR instrument (Bruker, AVANCE NEO 500, Germany).

#### 2.1.1 Preparation of single crystals

15 mg **F8·2HCl** was dissolved in acetone solvent and stirred at room temperature. During the stirring process, ethyl acetate solution was slowly added to make the mixed solvent reach the critical state of solid precipitation but quickly dissolved. The resulting solutions were then filtered through 0.45 µm nylon filters into clean vials and placed on a refrigerator**.** After standing for 3–10 days, small transparent crystals precipitated.

#### 2.1.2 HPLC

The final product, **F8·2HCl**, was tested for purity using a High Performance Liquid Chromatograph (Waterage, Alliance HPLC E2695, United States), and the purity of the product was above 98%. Agilent HC-C18 (2) 250 × 4.6 mm and 5 μm were used for the column. The chromatographic conditions were as follows: mobile phase was 70% chromatographic grade methanol and 30% water; detection wavelength was 254 nm; column temperature: 35°C; The flow rate was 1 mL/min.

### 2.2 Biologicals

#### 2.2.1 Cell lines and cell cultures

Undifferentiated human gastric cancer cell line (HGC-27) was purchased from the China Center for Type Collection (CCTCC, China) and used with 10% Foetal Bovine Serum (FBS, Gemini, Bio-Product, CA, United States), 1% penicillin streptomycin (PS) and 89% 1,640 medium (Eagle’s minimal essential medium) for culture. All cells were incubated at 37°C in a constant temperature incubator containing 95% air and 5% CO_2_.

#### 2.2.2 Cytotoxicity evaluation

To evaluate the cytotoxicity of **F8** and related organic salts on HGC-27 cells, MTT analysis was performed. HGC-27 cells were digested with trypsin, then cells were spread into 96-well plates at a density of 3,000 cells/well and incubated for 12–16 h. After 12 h, the medium in 96-well plates was replaced with compounds containing 1, 0.5, 0.25, 0.125, 0.062, 0.0312 μM for another 72 h. Three days later, 20 μL MTT solution was added and incubated at 37°C for 4 h. Then 150 μL DMSO solution was added to each well after the fluid was removed, and the absorbance was detected by microplate reader. The compound dissolved in DMSO was added to the cell medium so that the final concentration of DMSO was less than 0.4%.

#### 2.2.3 Animal experiments

All studies involving animals were conducted in accordance with the guidelines Animal Studies: Reports of *in vivo* Experiments, in accordance with ethics and were approved by the Institutional Ethics Committee of the University of South China (Hengyang, China). All animals (including KM mice, SD rats, and BALB/c nude mice) were purchased from Hunan Slake Jingda Laboratory Animal Ltd. (Changsha, China) and housed in gender-specific cages. The feeding environment is a specific pathogen free (SPF) level of 25°C constant temperature environment, 12 h of light and 12 h of night cycle alternated every day, the food and drinking water are through high pressure steam sterilization.

#### 2.2.4 Acute toxicity study

We investigated the toxicity of **F8·2HCl** when administered in three different ways, and **F8·2HCl** was all dissolved with normal saline, and the concentration of intravenous and intraperitoneal injection was 8 mg/mL, and the concentration of intragastric administration was 10 mg/mL. Ninety Kunming mice (45 males and 45 females) weighing 18–22 g were divided into 15 groups (*n* = 6). The doses of **F8·2HCl** injected by tail vein were 40 mg/kg, 60 mg/kg, 90 mg/kg, 110 mg/kg, 135 mg/kg, 200 mg/kg, 300 mg/kg, 400 mg/kg and 500 mg/kg, respectively. Intraperitoneal injection of different doses of **F8·2HCl** were 200 mg/kg, 250 mg/kg, 300 mg/kg, 360 mg/kg and 500 mg/kg, respectively. We also added a dose of 600 mg/kg administered by gavage. All drugs administered were dissolved in normal saline. Toxicity was monitored for 14 days. During the monitoring period, the status of mice was observed and their body weight was recorded.

#### 2.2.5 Pharmacokinetic studies

7–8-week Male SD rats weighing 180–220 g were allowed for this experiment. The rats were fasted 12 h except for water. The solution was administered to rats by intravenous injection of 10% ethanol +10% solutol as solvent and intragastric injection of 5% castor oil +5% solutol as solvent. The dose concentration was 8 mg/mL for intravenous injection and 10 mg/mL for gavage.

Twelve male SD rats were divided into 4 groups with 3 rats in each group. Group 1 was **F8**-intravenous injection group, the dose was 20 mg/kg; Group 2 was **F8**- intragastric administration group, the dose was 100 mg/kg; Group 3 was **F8·2HCl**-intragastric administration group, the dose was 100 mg/kg. Blank blood was collected before administration, and blood was collected at predetermined time points after administration:

Intravenous injection group: before administration, 2 min, 5 min, 15 min, 30 min, 1 h, 2 h, 4 h, 8 h, 12 h, 24 h. Intragastric administration group: Before administration, 5 min, 15 min, 30 min, 1 h, 2 h, 4 h, 6 h, 8 h, 12 h, 24 h. About 0.5 mL of blood was collected and placed in an EDTA-K2 tube at −80°C. The plasma was centrifuged and stored at −80°C. The samples were detected by liquid-mass spectrometry analysis system. The chromatographic column used was ODS-C18 (4.6 × 50 mm, 3 µm). The chromatographic conditions were as follows: Mobile phase A is aqueous phase, 5% water (including 0.1% formic acid); Mobile phase B is organic, 95% acetonitrile (containing 0.1% formic acid). Flow rate of 0.4 mL/min, column temperature: room temperature, according to the drug plasma concentration data, calculated pharmacokinetic parameters using DAS 2.0 software.

#### 2.2.6 Tumor xenograft model

4-week-old male BALB/c nude mice weighing 18g–22 g were injected with 100 μL HGC-27 cells (2 × 106 cells) to establish an undifferentiated gastric cancer model. After successful modeling, nude mice were randomly divided into five groups: blank group (*n* = 6), high-dose of **F8·2HCl** group (*n* = 6), low-dose of **F8·2HCl** group (*n* = 6), positive control drug apatinib group (*n* = 6) and **F8** group (*n* = 6). The high-dose group received 75 mg/kg and 37.5 mg/kg of **F8** hydrochloride, the control group received 37.5 mg/kg of apatinib and 75 mg/kg of **F8**, and the blank group received the same volume of normal saline. All drugs were dissolved in normal saline (5% DMSO and 5% castor oil were added to normal saline to facilitate the dissolution due to the poor water solubility of **F8**).

#### 2.2.7 Statistical analysis

GraphPad Prism 8.0 software was used for statistical analysis. Where two samples were compared, statistical significance was assessed using one-way analysis of variance (ANOVA). *p* < 0.05 was considered statistically significant.

## 3 Result and discussion

### 3.1 Synthesis route optimization

**SCHEME 1:**
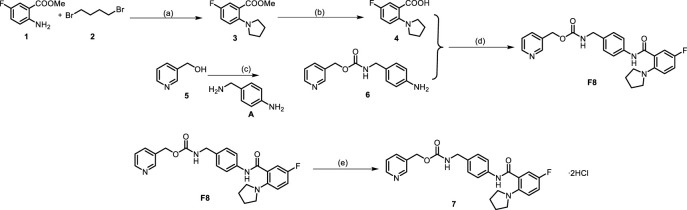
Synthetic route of compound **F8** without silica column chromatography purification. **(A)** Compound 1 (9 mmol), compound 2 (10.8 mmol), KI (19.8 mmol) and K_2_CO_3_ (19.8 mmol) in MeCN (10 mL) and 5% H_2_O was stirred at 90°C for 12 h, yield for 80%–90%; **(B)** Compound 3 (6 mmol) and 10% NaOH (5 mL) in EtOH (2 mL) was stirred at 85 °C for 3h, yield for 95%–100%; **(C)** Compound 5 (5 mmol), CDI (5.5 mmol), A (5 mmol), DBU (5 mmol), Et_3_N (7.5 mmol) in THF (20 mL) was stirred at room temperature, yield of up to 90%; **(D)** A mixture of Compound 4 (5 mmol), compound 6 (6 mmol), HATU (6 mmol), DIPEA (7.5 mmol) in DMF (20 mL) was stirred at room temperature, yield for 75%–90%. **(E)** The product **F8** (2 mmol) obtained in the previous step was dissolved with THF (10 mL), hydrogen chloride-methanol solution (5 mmol) was added, and the reaction was carried out at room temperature for half an hour after argon was introduced, yield for 95%–100%.

One step of the previous synthesis route of compound **F8** was the Ullmann coupling reaction, which had many defects such as the reaction quantity could not be amplified, the reaction conditions were harsh (strictly anhydrous and anaerobic), and the post-reaction treatment was difficult. Therefore, the previous synthesis route was not suitable for industrial production. At present, the synthetic route we have explored has many advantages, such as cheap raw materials and easy availability, mild reaction conditions at each step, simple post-processing, and green economy of the reaction system. The optimized synthetic route of **F8** is shown in Scheme 1, and the corresponding NMR data are presented in Supplementary Material S1.

### 3.2 Salt formation of compound **F8**

**FIGURE 2:**
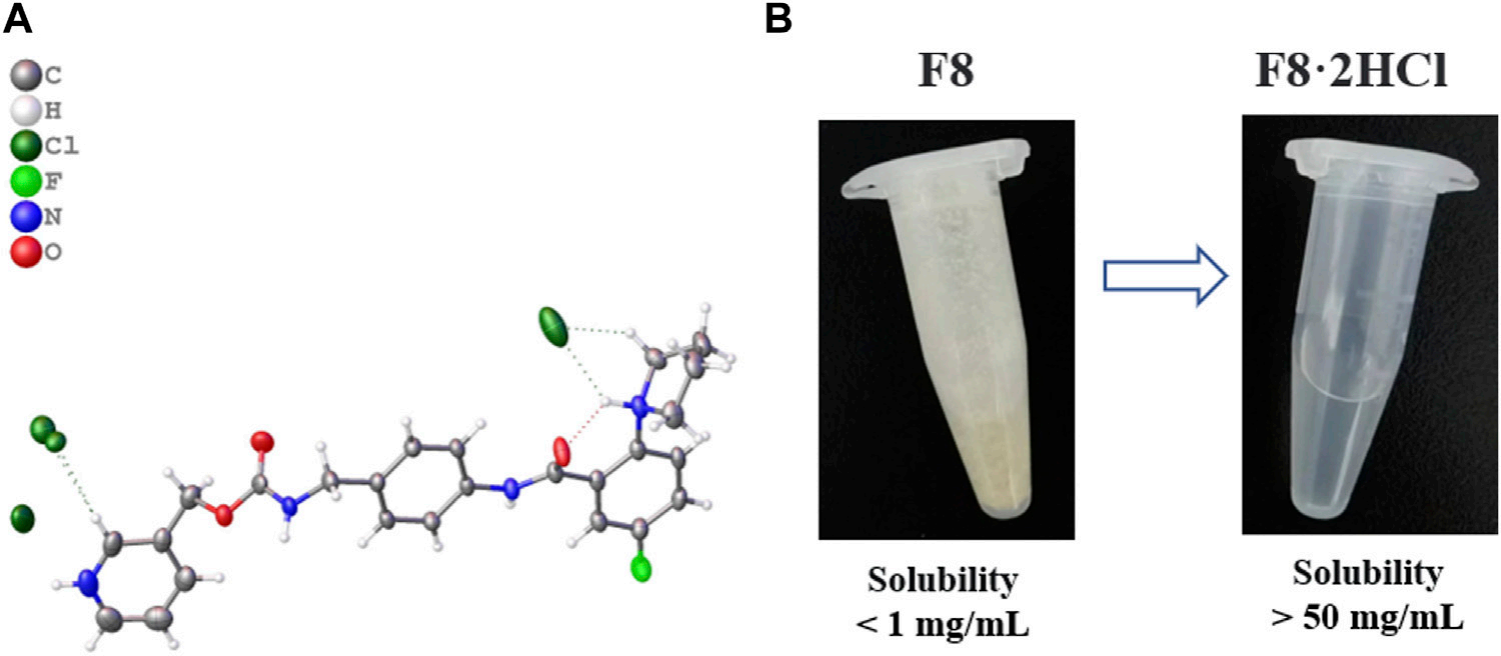
**(A)** Single crystal structure diagram of compound **F8·2HCl** (Deposition Number 2224701), compound **F8** combines 2 molecules of HCl to form **F8·2HCl**. **(B)** Solubility of compound **F8** and **F8·2HCl** in aqueous solution.

As mentioned earlier, in order to further solve the problems of poor solubility and poor oral absorption of compound **F8**, we considered to make **F8** into salt. The selection of salt form is a key step to the success of drug development, which requires a well-designed screening strategy to find the salt form that meets the target performance. Although there are many kinds of counter ions, the commonly used ones are very limited. We have tried some organic acids and inorganic acids respectively. Organic acids include L- (+) -tartaric, D- (+) -tartaric, citric, fumaric, oxalic, methyl-salicylic, maleic, L-melic, D-melic, benzenesulfonic, mesylate, succinic, adipic, inorganic acids including hydrochloric acid, hydrobromic acid and sulfuric acid, but most organic acids into salt have not succeeded, even though the reaction conditions were changed (temperature rise, water control and acid reaction rate improvement), they were still unsuccessful, only mesylate, hydrochloric acid, hydrobromic acid and sulfuric acid successfully prepared the corresponding organic salt (Table 1). This may be because the pKa value of these organic acids is relatively large, and it is not easy to form salt with **F8**. In order to get better salt-type compounds, we screened the best salt-type compounds from the aspects of hygroscopicity, solubility, irritability, stability and activity after salting. Finally, only mesylate, hydrochloric acid, hydrobromic acid and sulfuric acid have been successfully prepared into corresponding organic salts. We end up with these organic salts, them included **F8·2CH**_**3**_ **SO**_**2**_ **OH**, **F8·2HBr**, **F8·2HCl** and **F8·H**_**2**_ **SO**_**4**_. Firstly, **F8·2CH**_**3**_ **SO**_**2**_ **OH** has poor water solubility, and it is an oily, non-salt crystal. And **F8·2HBr** has strong hygroscopicity, it changed from a dry solid to a liquid state in just 4 h of exposure to the air, and it is highly irritating and harmful to human body. We also found that **F8·H**_**2**_ **SO**_**4**_ is less stable, oxidizing and turning black after a few hours of exposure to the air, which forced us to eliminate this organic salt. Finally, the best organic salt we get is **F8·2HCl**, it can remain stable under high temperature (80°C), acid, alkaline, oxidation and reduction conditions, and its solubility is improved greatly (Figure 2). The crystal data and structure refinement of **F8·2HCl** are shown in the Supplementary Material S2. And Chloride is the most common anion used to form weak basic drug salts. This is mainly due to the low pKa value of hydrochloric acid and its ability to easily form salts with weak bases.

**TABLE 1:** Screening of compound **F8** **related organic salts**.

Some acids	The corresponding organic salts	Crystalline	Hygroscopic	Solubility	Stability
L-tartaric	None	--	--	--	--
D-tartaric	None	--	--	--	--
Citric	None	--	--	--	--
Fumaric	None	--	--	--	--
Oxalic	None	--	--	--	--
Methyl-salicylic	None	--	--	--	--
Maleic	None	--	--	--	--
L-melic	None	--	--	--	--
D-melic	None	--	--	--	--
Benzenesulfonic	None	--	--	--	--
Succinic	None	--	--	--	--
Adipic	None	--	--	--	--
Mesylate	F8·2CH_3_SO_2_OH	None, oily substance	--	--	--
Hydrobromic acid	F8·2HBr	crystallization	Strong moisture absorption	--	--
Sulfuric acid	F8·H_2_SO_4_	crystallization	Almost no moisture absorption	Soluble	Easily oxidized in air
Hydrochloric acid	F8·2HCl	crystallization	Almost no moisture absorption	Soluble	Keep stable in extreme environment

After compound **F8** was salted, its water solubility was greatly improved, and its solubility in pure water increased from less than 1 mg/mL to more than 50 mg/mL. Considering that the subsequent experiments *in vivo* all used normal saline as solvent, we further investigated the solubility of compounds **F8** and **F8·2HCl** in normal saline, and found that the results were not much different from pure water.

### 3.3 Evaluation of activity *in vitro*

Infinite proliferation is one of the most prominent features of cancer cells, therefore, we evaluated the anti-proliferation activity of compounds **F8** and related organic salts against human gastric carcinoma HGC-27 via MTT assay. The results showed that **F8** and its salt compounds had good anti-proliferation activity against human undifferentiated cells HGC-27 and liver cancer cells, as well as breast cancer cells MDA-MB-468. The IC_50_ value at 72 h was less than 1.5 μM, indicating that the compounds had certain anti-proliferation ability against these cancer cells (Table 2). Although the activity of compound **F8·2HCl** compared to **F8** was not significantly improved *in vitro*, which may be due to the fact that salt formation did not change the activity of compound **F8**, instead retained its anticancer activity, and promoted its absorption *in vivo* by improving its water solubility, thus improving its efficacy *in vivo*.

**TABLE 2:** Effect of compound **F8** **and organic salts on cancer cell viability**.

IC_50_(μmol)	F8	F8·2HCl	F8·H_2_SO_4_
HGC-27	0.26 ± 0.03	0.25 ± 0.01	0.29 ± 0.03
MDA-MB-468	1.45 ± 0.12	1.42 ± 0.19	1.46 ± 0.08
SK-Hep-1	1.65 ± 0.15	9.37 ± 0.91	—
HuH7	2.35 ± 0.29	10.90 ± 0.97	—
HCT-116	4.89 ± 0.35	4.02 ± 0.28	7.74 ± 0.07
HCC-1806	21.13 ± 0.74	16.17 ± 0.41	27.33 ± 0.81
MCF-7	43.50 ± 0.36	47.69 ± 0.15	49.19 ± 0.37
HCC-1937	47.05 ± 0.07	48.81 ± 0.18	84.77 ± 0.38
MDA-MB-231	53.76 ± 0.59	47.01 ± 0.62	51.16 ± 0.49

#### 3.3.1 *In vivo* acute toxicity assay

To evaluate the safety of **F8·2HCl** *in vivo*, acute toxicity studies were performed. First, six Kunming mice (half male and half female) were gavage with **F8·2HCl** at a single dose of 600 mg/kg. All the mice survived 14 days without any abnormal reactions. The same 6 KM mice were intraperitoneally injected with different doses of **F8·2HCl**, and the LD50 obtained of intraperitoneal injection of **F8·2HCl** was determined to be 303 mg/kg. Under the same conditions for intravenous injection of **F8·2HCl**, its LD_50_ is 153 mg/kg. In addition, HE staining of pathological section proved that the structure of the heart, liver, spleen, lung, kidney and other organs was intact without any lesions at the doses of 200 mg/kg intraperitoneal injection and 600 mg/kg oral administration.

#### 3.3.2 Preliminary pharmacokinetic properties of compounds F8 and F8·2HCl

**FIGURE 3:**
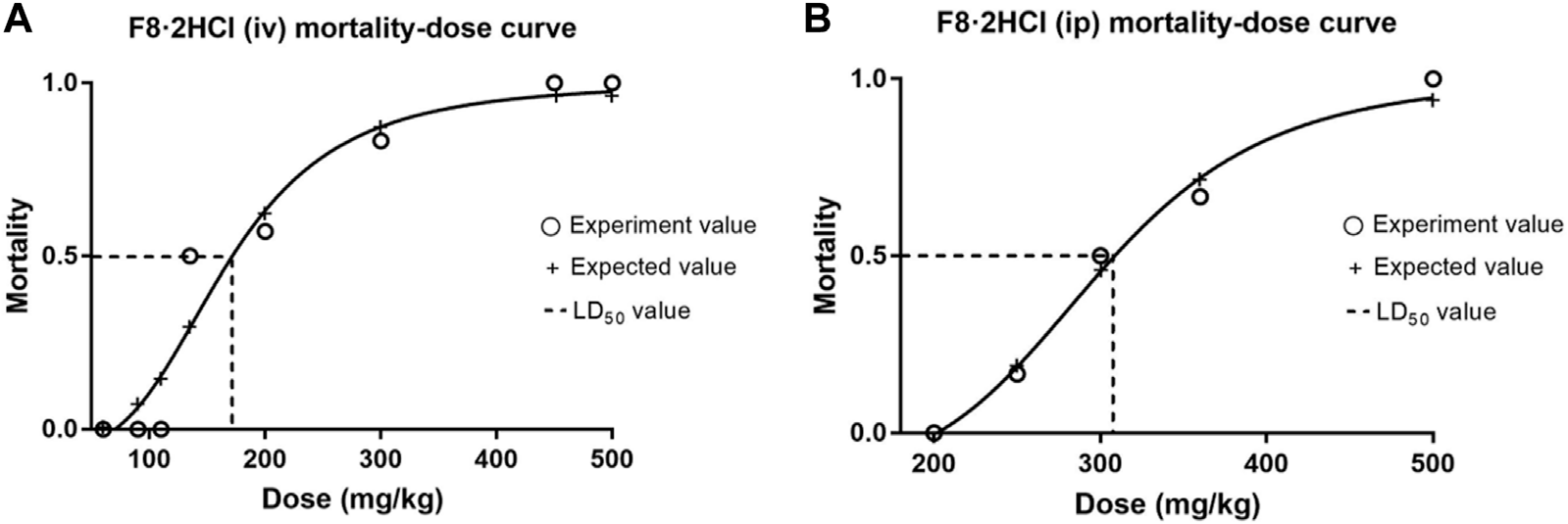
The mortality-dose curves of **F8·2HCl**. **(A)** The LD_50_ was found to be 153 mg/kg when administered intravenously at different doses. **(B)** The LD_50_ was found to be 303 mg/kg when administered intraperitoneally at different doses.

**FIGURE 4:**
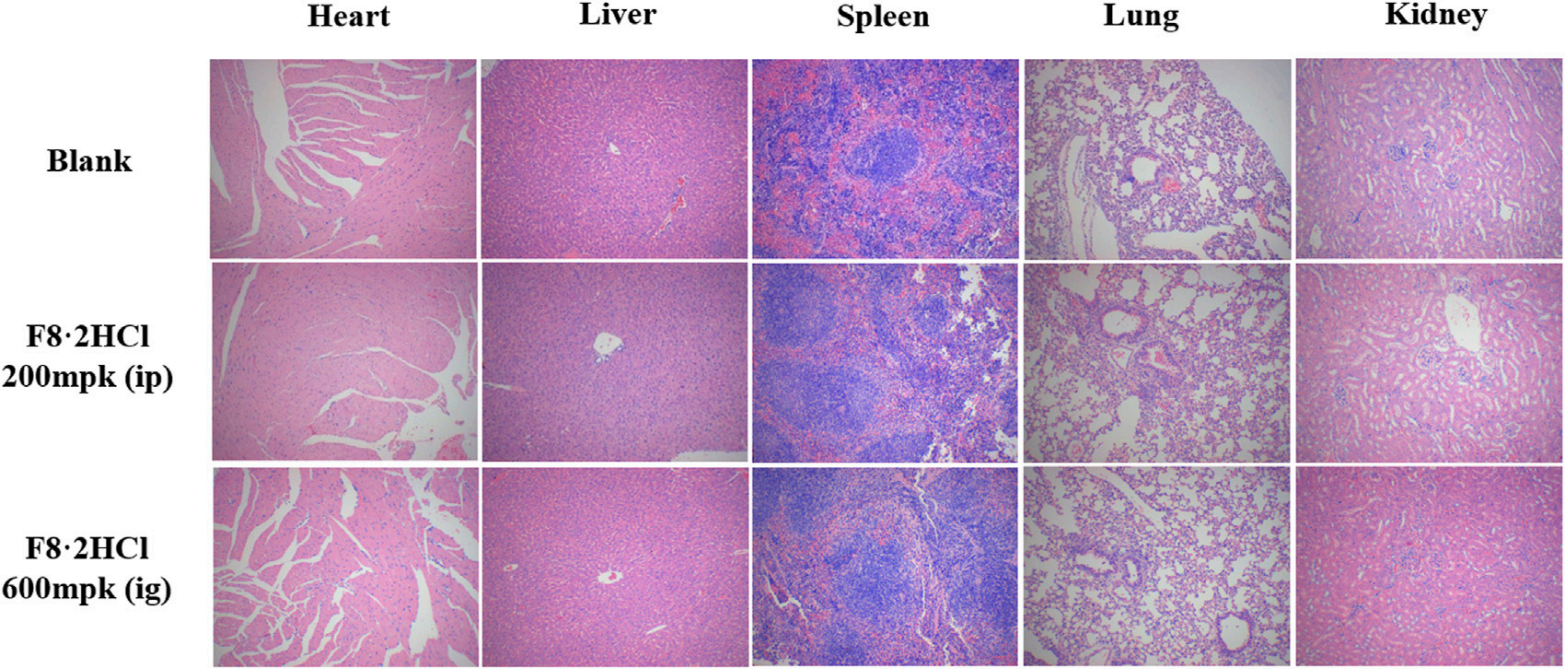
HE staining of the heart, liver, spleen, lung, and kidney from animals treated with compound **F8·2HCl** (200 mg/kg through intraperitoneal injection and 600 mg/kg through intragastric administration) or equal solution without compounds. All images are viewed at 100x.

After oral administration, drugs need to go through four stages, namely, absorption, distribution, metabolism and excretion. However, the changes in solubility of drugs after salting only affect the absorption stage. Since absorption is accompanied by a dissolution process, the water solubility and dissolution rate of drugs are the main factors that determine their absorption, and then affect their oral bioavailability (Elder et al., 2013). Under the stimulation of cell activity and increased solubility, pharmacokinetic experiments were carried out to understand the drug exposure and provide a basis for further druggability of compound **F8**. In this study, compound **F8** and **F8·2HCl** were administered in two different ways: tail vein injection (20 mg/kg) and gavage (100 mg/kg), and pharmacokinetic parameters were measured in Sprague-Dawley (SD) rats (Figures 3, 4).

From Table 3, the area under the concentration-time curve (AUC_0-t_) of compounds **F8** in intravenous injection and oral administration was about 6.99 mg·h/mL and 1.13 mg·h/mL, while the AUC0-t of compound **F8·2HCl** was 1.77 mg·h/mL. Besides, the plasma concentration of compound **F8** reached a peak of 0.4 mg/mL at 0.19 h, while 0.58 mg/mL at 1.42 h for compound **F8·2HCl**. Additionally, the half-life (T_1/2_) was 3.63 h for compound **F8·2HCl**, whereas the parameters were shorter for compound **F8** at 2.66 h, and T_max_ has been lengthened nearly tenfold. And bioavailability was also increased from 3.23% of **F8** to 5.06% of **F8·2HCl**. In conclusion, compound **F8·2HCl** not only has a higher blood concentration than compound **F8**, but also has a longer half-life of action, so it is highly likely that its drug effect *in vivo* is more significant than that of compound **F8**.

**TABLE 3:** *In vivo* pharmacokinetic properties of compounds **F8** **and** **F8·2HCl**.

Compound	Dose (mg/kg)	Route	AUC (0-∞) (μg/mL*h)	C_max (_μg/mL)	T_1/2_ (h)	T_max_ (h)
F8	20	iv	6.99	12.10	3.09	0.03
F8	100	ip	1.13	0.40	2.66	0.19
F8·2HCl	100	ip	1.77	0.58	3.63	1.42

#### 3.3.3 *In vivo* anti-HGC-27 xenograft activity of F8·2HCl

**FIGURE 5:**
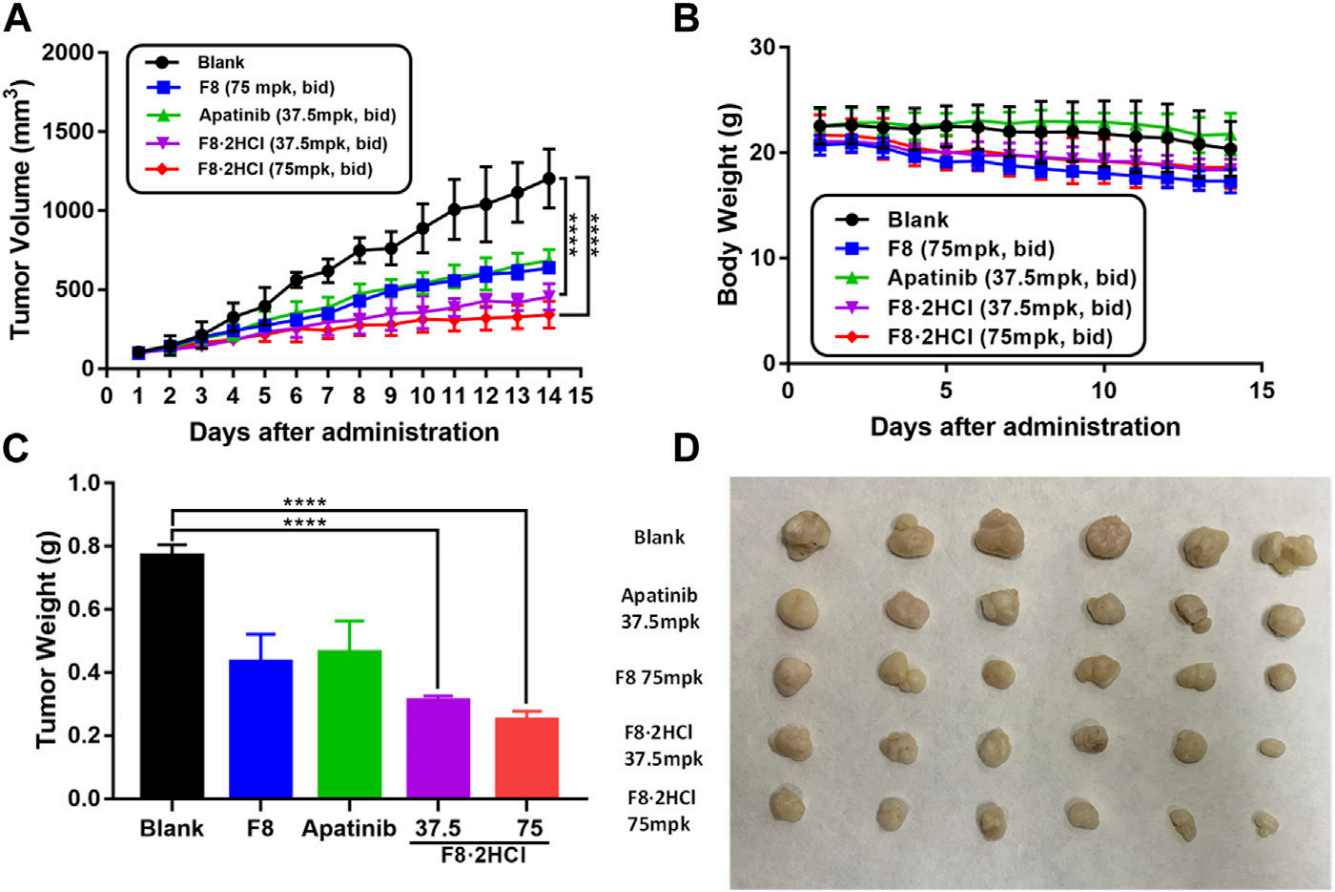
Compound **F8·2HCl** suppressed gastric xenograft tumor growth *in vivo*. Male BALB/c nude mice were divided into five groups (*n* = 6 per group) including vehicle group, apatinib (positive drug) at 37.5 mg/kg dose, compound **F8·2HCl** at 37.5 mg/kg dose, compound **F8·2HCl** at 75 mg/kg dose and administrated via oral gavage twice daily for 15 days. **(A)** Tumor volumes plotted as mean ± SEM. **(B)** Mice weight curve during treatment plotted as mean ± SD. **(C,D)** Tumor weight and size on the final day. Statistical analyses showed the mean ± SD or SEM of three independent experiments, comparing each treatment group to the blank and apatinib group: (*) *p* < 0.05, (**) *p* < 0.01, (***) *p* < 0.001.

Encouraged by the cellular activities and drug metabolism in rat, we evaluated the *in vivo* anti-gastric tumor potency of compound **F8·2HCl** on HGC-27 xenograft model using the clinical third-line oral anti-gastric cancer agent apatinib and the prototype drug **F8** as the positive control. As shown in Table 4, after continuous administration by oral administration for 14 days, all compounds showed a slow growth trend on tumor volume, but compound **F8·2HCl** had a more significant inhibitory effect than the prototype compound **F8** and the marketed drug apatinib. The tumor growth inhibition rate of compound **F8·2HCl** was 70.1% at a dose of 75 mg/kg and 60.6% at a dose of 37.5 mg/kg, all of which outdistanced a 52.8% and 50.0% inhibition of compound **F8** and apatinib at a dose of 75 mg/kg and 37.5 mg/kg. So, it means that the tumor growth inhibition rates of two different doses of **F8·2HCl** both exceeded compounds **F8** and apatinib at the same dose. Similarly, treatment with compound **F8·2HCl** resulted in a significant decrease in tumor weight compared with the control group in a concentration dependent manner. Importantly, no significant weight loss was observed compared to the control group, indicating that compound **F8·2HCl** had no significant adverse events in mice (Figure 5).

**TABLE 4:** Summary of tumor growth inhibition of compound **F8**, **F8·2HCl** **and apatinib**.

Compound	Administration		Dose (mg/kg)	Inhibition (%)
	Schedule	Route		
F8	p.o.	bid	75.0	52.8
Apatinib	p.o.	bid	37.5	50.0
F8·2HCl	p.o.	bid	37.5	60.6
	p.o.	bid	75.0	70.1

**FIGURE 6:**
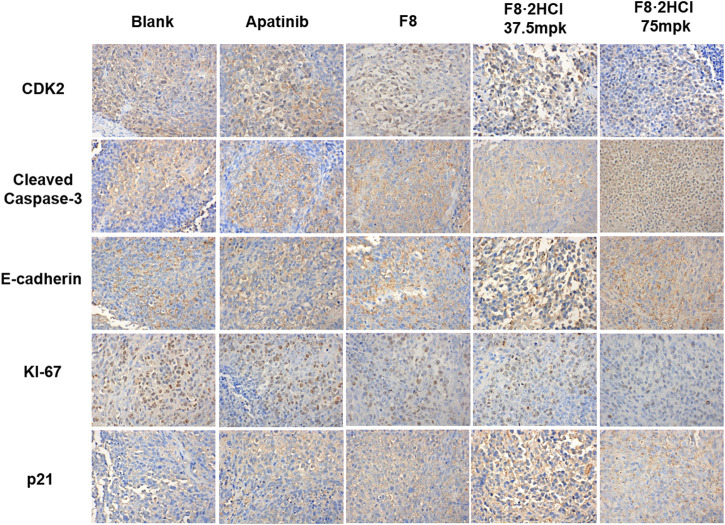
Compound **F8·2HCl** regulated apoptosis-related proteins and cell cycle-related proteins *in vivo*. Representative tumor images of IHC analysis stained by CDK2, Cleaved Caspase-3, E-cadherin, KI-67 and p21. Magnification: × 400.

Then, immunohistochemical analysis was performed on the tumor of the HGC-27 xenograft tumor model. It is known that cyclin-dependent kinase 2 (CDK2) and CDK inhibitor p21 play pivotal parts in cell cycle regulation, while both downregulation of CDK2 or upregulation of p21 lead to cell cycle arrest (Tadesse et al., 2019; Sturmlechner et al., 2021). Compared with the blank group, the expression of CDK2 declined dose-dependently after treatment of compound **F8·2HCl**, while the level of p21 protein was significantly upregulated (Figure 6). Ki-67 is an antigen associated with proliferating cells, which is usually positively correlated with tumor growth rate (Menon et al., 2019). It is found that compound **F8·2HCl** significantly decreased the expression of Ki-67 protein. Cleaved Caspase-3 is a pro-apoptotic protein, the higher the expression of this protein, the stronger the ability of rapid proliferation of cancer cells (Wei et al., 2020). As shown in Figure 6, **F8·2HCl** significantly upregulated the expression of Cleaved Caspase-3 protein. E-cadherin which is a type of cell surface transmembrane glycoprotein has an important influence on the cell-cell adhesion function. Growing evidences suggested that E-cadherin is a critical tumor suppressor in several carcinomas, including gastric cancer, and the decreased expression of E-cadherin was mainly found in diffuse type gastric cancer (Zhao et al., 2021). It is also confirmed that **F8·2HCl** can increase the expression level of E-cadherin protein, compared with the blank group. These results suggest that compound **F8·2HCl** exerts anticancer activity by regulating cycle-, apoptosis-, and cell adhesion-related proteins.

Therefore, although **F8·2HCl** did not improve the activity *in vitro*, it greatly improved the absorption of the drug in the body and promoted the play of the drug effect. Perhaps we can draw a conclusion that compound **F8·2HCl** can improve the exposure level of drugs in the body by improving the solubility and dissolution rate of compound **F8**, so as to improve the absorption, distribution and metabolism of drugs, and finally achieve the purpose of enhancing the druggability.

## 4 Conclusion

In summary, on the basis of the previous work, we found the compound **F8** as an early preclinical candidate for treatment of gastric cancer. Nevertheless, the poor solubility of compound **F8** hinders its further clinical research. Therefore, we applied salt formation method to improve the water solubility and oral exposure of compound **F8.** And our continuous efforts have finally confirmed **F8·2HCl** salt form with maintained *in vitro* antitumor activity, improved water solubility and pharmacokinetic properties.

The anticancer activity of compound **F8·2HCl** was not significantly improved *in vitro*, which may be due to the fact that salt formation only changed its physicochemical properties, but did not change the pharmacological activity of compound **F8**. Additionally, compounds **F8** and **F8·2HCl** exert broad-spectrum anticancer properties, but also exhibit obvious selectivity to different cancer cells. This selectivity of **F8** and **F8·2HCl** against cancer cells might be caused by many aspects. Given that compound **F8** could regulate the cell cycle-related proteins and induce cell cycle arrest at G2/M phase, the different differentiation and proliferation rate of cancer cells affects their sensitivity to **F8** and **F8·2HCl**. In our previous work, compound **F8** had exhibited high selectivity for malignant over normal cells (Feng et al., 2022), with excellent potential on undifferentiated gastric cancer cell (IC_50_ = 0.28 μM for HGC-27) than differentiated gastric cancer cells (IC_50_ = 10.66 μM and 23.29 μM for MGC-803 and AGS, respectively) and normal cells (IC_50_ > 100 μM for GES-1). On the other hand, it is speculated that the expressed difference of CDK16 in different cancer cells might affect the selectivity of **F8** and **F8·2HCl** against cancer cells. It is known that CDK16 plays an important role in the development and progression of various cancer cells, such as breast cancer cell MDA-MB-468, colon cancer cell HCT-116, prostatic cancer cell DU-145, etc., rather than breast cancer cell MCF-7 (Yanagi et al., 2014; Yanagi et al., 2016). CDK16 had been identified as a potential target of compound **F8** by activity-based protein analysis technique in the previous study. Consistently, compound **F8** and **F8·2HCl** exhibited potent inhibitory effects on MDA-MB-468 (IC_50_ = 1.45 μM and 1.42 μM for **F8** and **F8·2HCl**) and HCT-116 (IC_50_ = 4.89 μM and 4.02 μM for **F8** and **F8·2HCl**), rather than MCF-7 (IC_50_ = 43.50 μM and 47.69 μM for **F8** and **F8·2HCl**). Nevertheless, these still need to be confirmed by further studies.

Drugs with suboptimal aqueous solubility often have low and variable oral bioavailability, which might account for their unpredictable clinical response. Improving the aqueous solubility of candidate drugs through salt formation, thereby improving their pharmacokinetic properties and efficacy *in vivo*, has become a feasible means in the early stage of drug development. The results confirmed that the water solubility of **F8·2HCl** was more than 50 times higher than that of compound **F8**, which greatly promoted the pharmacokinetic properties of **F8·2HCl** *in vivo*. Even with the same oral dose, the mean serum concentrations and maximum serum concentrations in the **F8·2HCl** group were higher than those in the **F8** group, and the half-life was extended by 1 h, and the T_max_ increased nearly 10 times as much. *In vivo* anti-xenograft tumor experiment, the **F8·2HCl** (TGI of 70.1% in 75 mg/kg) displayed more superior *in vivo* antitumor efficacy than compound **F8** (TGI of 52.8% in 75 mg kg), and the **F8·2HCl** (TGI of 60.6% in 37.5 mg/kg) also showed better than apatinib (TGI of 50.0% in 37.5 mg/kg). Moreover, the further immunohistochemical analysis revealed that **F8·2HCl** exerts a pro-apoptotic effect through the regulation of cell cycle and apoptosis -related proteins. **F8·2HCl** significantly decreased the expression of CDK2 and KI-67 protein, and increased the expression of Cleaved Caspase3 and E-cadherin. The specific mechanism of action of compound **F8·2HCl** has been further investigated, and the preclinical studies of **F8·2HCl** are under way.

In conclusion, compound **F8·2HCl** showed excellent activity *in vivo*, which also indicated that good water solubility was conducive to the play of drug efficacy and the enhancement of drug-like properties of the compound, as well as to improve the pharmacokinetic characteristics of drug absorption, distribution, metabolism and excretion (ADME) in body. Therefore, salting is an effective means to improve the drug-like properties of compound **F8**, and **F8·2HCl** can serve as a promising therapeutic agent against undifferentiated gastric cancer.

## Data Availability

The original contributions presented in the study are included in the article/Supplementary Material, further inquiries can be directed to the corresponding authors.
