# Supplementary material for: Development of o-aminobenzamide salt derivatives for improving water solubility and anti-undifferentiated gastric cancer
Source: Front Pharmacol. 2023 Jul 11;14:1118397. doi: 10.3389/fphar.2023.1118397 (PMC10368370; doi:10.3389/fphar.2023.1118397)
Supplement: Supplementary file 1 [file DataSheet2.pdf]

Supplementary Material File 2：Crystal data and structure refinement for F8·2HCl

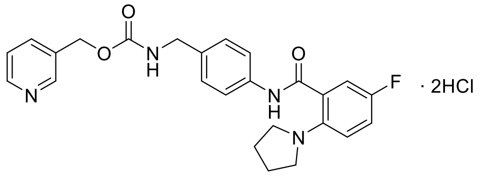

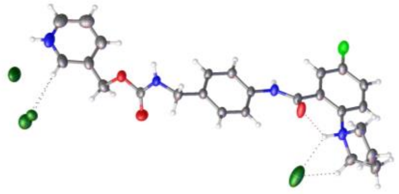

Table S1 Crystal data and structure refinement for F8·2HCl.

Identification code	F8·2HCl
Empirical formula	$C_{25}H_{27}Cl_2FN_4O_3$
Formula weight	521.40
Temperature/K	170.00
Crystal system	triclinic
Space group	P-1
a/Å	9.7804(10)
b/Å	10.9024(11)
c/Å	13.6391(14)
α/°	102.401(4)
β/°	99.217(4)
γ/°	99.557(4)
Volume/Å^3	1371.4(2)
Z	2
ρ_{calc}g/cm^3	1.263
μ/mm^{-1}	1.620
F(000)	544.0
Crystal size/mm^3	0.15 × 0.08 × 0.05
Radiation	GaKα (λ = 1.34139)
2Θ range for data collection/°	8.146 to 107.8
Index ranges	-11 ≤ h ≤ 11, -13 ≤ k ≤ 13, -16 ≤ l ≤ 16
Reflections collected	13983
Independent reflections	4970 [R_{int} = 0.0445, R_{sigma} = 0.0504]
Data/restraints/parameters	4970/1/338
Goodness-of-fit on F^2	1.036
Final R indexes [I>=2σ (I)]	R_1 = 0.0718, wR_2 = 0.1944
Final R indexes [all data]	R_1 = 0.0799, wR_2 = 0.2001
Largest diff. peak/hole / e Å^{-3}	1.15/-0.47

Table S2 Fractional Atomic Coordinates (×10^4) and Equivalent Isotropic Displacement Parameters (Å^2×10^3) for F8·2HCl. U_{eq} is defined as 1/3 of the trace of the orthogonalised U_{IJ} tensor.

Atom	*x*	*y*	*z*	U(eq)
F1	9120(2)	8669(2)	13135.6(14)	49.2(5)
O1	4661(2)	6451(2)	3914.7(16)	36.2(5)
O2	6665(3)	5690(2)	4197(2)	52.9(7)
O3	12101(2)	9083(2)	9766.7(16)	41.4(6)
N1	121(4)	5022(3)	2217(3)	53.0(8)
N2	6639(3)	7793(2)	4760.0(18)	32.6(6)
N3	9758(3)	8165(2)	9434.7(18)	28.7(5)
N4	13729(3)	8400(2)	11252.7(19)	32.0(6)
C1	-492(5)	5347(4)	3006(4)	55.6(10)
C2	291(5)	5659(4)	3977(3)	53.7(10)
C3	1714(4)	5605(3)	4129(3)	45.5(8)
C4	2344(4)	5237(3)	3296(2)	37.4(7)
C5	1493(4)	4949(3)	2332(3)	44.6(8)
C6	3868(4)	5164(3)	3439(3)	41.3(8)
C7	6064(3)	6586(3)	4295(2)	32.3(7)
C8	8144(3)	8218(3)	5211(2)	34.6(7)
C9	8542(3)	8145(3)	6321(2)	28.0(6)
C10	7580(3)	8181(3)	6967(2)	29.8(6)
C11	8000(3)	8201(3)	7996(2)	27.6(6)
C12	9399(3)	8177(3)	8387(2)	25.9(6)
C13	10366(3)	8114(3)	7736(2)	28.0(6)
C14	9936(3)	8105(3)	6716(2)	29.5(6)
C15	11050(3)	8607(3)	10049(2)	29.2(6)
C16	11170(3)	8507(3)	11143(2)	27.6(6)
C17	10033(3)	8575(3)	11637(2)	31.5(7)
C18	10220(3)	8573(3)	12658(2)	32.6(7)
C19	11477(3)	8490(3)	13224(2)	32.7(7)
C20	12616(3)	8417(3)	12738(2)	31.6(7)
C21	12464(3)	8436(3)	11714(2)	28.4(6)
C22	14813(4)	9664(3)	11610(3)	44.1(8)
C23	16228(4)	9262(4)	11658(3)	49.0(9)
C24	15989(4)	8060(4)	12043(3)	45.4(8)
C25	14537(3)	7357(3)	11450(3)	39.0(7)
Cl2	2992(4)	3708(8)	290(3)	33.8(12)
Cl3	1866.1(18)	5675.0(16)	-302.2(15)	73.7(7)
Cl1	14313.3(18)	7222(2)	8805.2(16)	83.2(8)

Table S2 Fractional Atomic Coordinates (×10^4) and Equivalent Isotropic Displacement Parameters (Å^2×10^3) for F8·2HCl. U_{eq} is defined as 1/3 of the trace of the orthogonalised U_{IJ} tensor.

Atom	*x*	*y*	*z*	U(eq)
Cl4	2767(11)	3120(30)	92(12)	56(3)

Table S3 Anisotropic Displacement Parameters (Å^2×10^3) for F8·2HCl. The Anisotropic displacement factor exponent takes the form: $-2\pi^2[h^2a^{*2}U_{11}+2hka^*b^*U_{12}+\ldots]$.

Atom	U_{11}	U_{22}	U_{33}	U_{23}	U_{13}	U_{12}
F1	37.3(11)	81.0(15)	31.2(10)	18.5(10)	9.0(8)	9.9(10)
O1	37.3(12)	33.2(11)	33.1(12)	6.2(9)	-3.7(9)	6.2(9)
O2	58.1(16)	41.2(14)	55.0(16)	4.3(11)	-4.0(12)	23.2(12)
O3	29.2(11)	61.4(15)	29.3(11)	23.0(10)	-2.0(9)	-10.3(10)
N1	56(2)	33.4(15)	56(2)	7.2(14)	-16.0(16)	2.4(14)
N2	34.1(14)	36.8(14)	24.7(12)	6.1(10)	-4.2(10)	12.7(11)
N3	26.3(12)	33.9(13)	24.4(12)	11.8(10)	1.0(10)	0.0(10)
N4	27.3(13)	36.6(14)	29.6(13)	13.7(11)	-1.2(10)	-0.3(10)
C1	53(2)	35.3(19)	76(3)	17.3(19)	2(2)	7.6(17)
C2	64(3)	46(2)	63(3)	27.2(19)	23(2)	17.4(18)
C3	59(2)	42.6(19)	36.1(18)	15.5(15)	4.7(16)	10.2(17)
C4	48.3(19)	26.7(15)	32.8(17)	9.5(12)	-0.1(14)	0.7(13)
C5	59(2)	28.8(16)	37.4(19)	3.2(13)	-2.3(16)	1.7(15)
C6	52(2)	31.1(16)	36.7(18)	5.5(13)	4.2(15)	4.0(14)
C7	40.4(17)	39.1(17)	19.1(14)	10.2(12)	0.7(12)	14.2(14)
C8	33.4(16)	44.9(18)	25.4(15)	11.7(13)	1.6(12)	8.5(14)
C9	30.8(15)	30.4(15)	22.3(14)	7.7(11)	2.2(11)	6.5(12)
C10	24.7(14)	34.4(15)	27.5(15)	8.4(12)	-2.3(11)	4.7(12)
C11	24.3(14)	32.0(15)	26.5(14)	9.1(12)	5.3(11)	2.8(11)
C12	27.2(14)	24.7(13)	23.0(14)	8.6(11)	-2.0(11)	1.1(11)
C13	22.4(14)	31.6(15)	27.4(15)	7.9(12)	-1.2(11)	3.5(11)
C14	27.8(15)	34.7(15)	25.4(15)	8.4(12)	5.0(12)	3.8(12)
C15	27.7(15)	30.7(15)	26.9(15)	11.1(12)	-0.5(12)	-0.1(12)
C16	27.6(14)	24.3(14)	27.4(15)	9.1(11)	-0.1(11)	-2.3(11)
C17	26.2(15)	35.9(16)	29.8(16)	10.6(12)	0.1(12)	1.0(12)
C18	31.3(16)	37.5(16)	27.0(15)	9.6(12)	4.9(12)	0.8(13)
C19	39.7(17)	33.9(16)	20.3(14)	7.6(12)	0.5(12)	0.2(13)
C20	30.1(15)	32.4(15)	27.2(15)	9.3(12)	-5.2(12)	0.3(12)
C21	28.7(15)	26.7(14)	26.9(15)	10.2(11)	-0.5(12)	-1.4(11)
C22	37.9(18)	40.9(18)	48(2)	16.1(15)	2.4(15)	-9.4(15)

Table S3 Anisotropic Displacement Parameters ($Å^2×10^3$) for F8·2HCl. The Anisotropic displacement factor exponent takes the form: $-2\pi^2[h^2a^{*2}U_{11}+2hka^*b^*U_{12}+…]$.

Atom	U_{11}	U_{22}	U_{33}	U_{23}	U_{13}	U_{12}
C23	31.2(18)	65(2)	44(2)	15.0(17)	2.9(15)	-8.4(16)
C24	31.9(17)	70(2)	34.0(18)	18.6(16)	0.3(14)	8.0(16)
C25	30.8(16)	46.7(19)	43.7(19)	19.7(15)	7.2(14)	9.3(14)
Cl2	30.5(13)	34(2)	36.2(13)	7.1(13)	12.3(8)	0.9(12)
Cl3	55.6(10)	56.0(10)	81.4(13)	-14.3(8)	-18.2(8)	5.6(7)
Cl1	54.4(10)	102.7(15)	94.1(14)	63.4(12)	-8.6(9)	-7.0(9)
Cl4	47(4)	55(10)	61(4)	16(5)	8(3)	-1(4)

Table S4 Bond Lengths for F8·2HCl.

Atom	Atom	Length/Å	Atom	Atom	Length/Å
F1	C18	1.352(4)	C4	C6	1.489(5)
O1	C6	1.442(4)	C8	C9	1.525(4)
O1	C7	1.359(4)	C9	C10	1.387(4)
O2	C7	1.215(4)	C9	C14	1.395(4)
O3	C15	1.229(4)	C10	C11	1.392(4)
N1	C1	1.325(6)	C11	C12	1.394(4)
N1	C5	1.344(5)	C12	C13	1.397(4)
N2	C7	1.316(4)	C13	C14	1.384(4)
N2	C8	1.456(4)	C15	C16	1.507(4)
N3	C12	1.419(4)	C16	C17	1.394(4)
N3	C15	1.348(4)	C16	C21	1.399(4)
N4	C21	1.477(4)	C17	C18	1.375(4)
N4	C22	1.524(4)	C18	C19	1.371(4)
N4	C25	1.534(4)	C19	C20	1.389(5)
C1	C2	1.361(6)	C20	C21	1.385(4)
C2	C3	1.387(6)	C22	C23	1.516(5)
C3	C4	1.394(5)	C23	C24	1.511(5)
C4	C5	1.380(5)	C24	C25	1.504(5)

Table S5 Bond Angles for F8·2HCl.

Atom	Atom	Atom	Angle/°	Atom	Atom	Atom	Angle/°
C7	O1	C6	116.8(2)	C11	C12	N3	117.5(3)
C1	N1	C5	122.6(3)	C11	C12	C13	119.5(3)
C7	N2	C8	122.2(3)	C13	C12	N3	123.0(2)

Table S5 Bond Angles for F8·2HCl.

Atom	Atom	Atom	Angle/°	Atom	Atom	Atom	Angle/°
C15	N3	C12	126.1(2)	C14	C13	C12	119.9(3)
C21	N4	C22	112.9(3)	C13	C14	C9	121.0(3)
C21	N4	C25	115.2(2)	O3	C15	N3	123.8(3)
C22	N4	C25	106.1(2)	O3	C15	C16	119.6(3)
N1	C1	C2	119.6(4)	N3	C15	C16	116.5(2)
C1	C2	C3	119.5(4)	C17	C16	C15	121.3(3)
C2	C3	C4	120.5(3)	C17	C16	C21	118.1(3)
C3	C4	C6	121.5(3)	C21	C16	C15	120.5(3)
C5	C4	C3	116.9(3)	C18	C17	C16	119.3(3)
C5	C4	C6	121.5(3)	F1	C18	C17	118.8(3)
N1	C5	C4	120.8(4)	F1	C18	C19	117.9(3)
O1	C6	C4	106.4(3)	C19	C18	C17	123.3(3)
O2	C7	O1	122.8(3)	C18	C19	C20	117.9(3)
O2	C7	N2	126.6(3)	C21	C20	C19	120.1(3)
N2	C7	O1	110.5(3)	C16	C21	N4	121.2(2)
N2	C8	C9	114.5(3)	C20	C21	N4	117.4(3)
C10	C9	C8	122.2(3)	C20	C21	C16	121.3(3)
C10	C9	C14	118.8(3)	C23	C22	N4	104.1(3)
C14	C9	C8	118.9(3)	C24	C23	C22	103.2(3)
C9	C10	C11	120.8(3)	C25	C24	C23	103.3(3)
C10	C11	C12	120.0(3)	C24	C25	N4	105.7(3)

Table S6 Torsion Angles for F8·2HCl.

A	B	C	D	Angle/°	A	B	C	D	Angle/°
F1	C18	C19	C20	-178.7(3)	C10	C11	C12	C13	-1.0(4)
O3	C15	C16	C17	149.0(3)	C11	C12	C13	C14	1.5(4)
O3	C15	C16	C21	-26.5(4)	C12	N3	C15	O3	-0.2(5)
N1	C1	C2	C3	-1.4(5)	C12	N3	C15	C16	179.2(3)
N2	C8	C9	C10	-24.5(4)	C12	C13	C14	C9	-0.7(4)
N2	C8	C9	C14	158.7(3)	C14	C9	C10	C11	1.2(4)
N3	C12	C13	C14	178.8(3)	C15	N3	C12	C11	-153.7(3)
N3	C15	C16	C17	-30.4(4)	C15	N3	C12	C13	28.9(4)
N3	C15	C16	C21	154.1(3)	C15	C16	C17	C18	-175.4(3)
N4	C22	C23	C24	-37.2(3)	C15	C16	C21	N4	-2.6(4)
C1	N1	C5	C4	-1.2(5)	C15	C16	C21	C20	176.3(3)
C1	C2	C3	C4	0.1(5)	C16	C17	C18	F1	178.5(3)
C2	C3	C4	C5	0.6(5)	C16	C17	C18	C19	-0.8(5)

Table S6 Torsion Angles for F8·2HCl.

A	B	C	D	Angle/°	A	B	C	D	Angle/°
C2	C3	C4	C6	-179.6(3)	C17	C16	C21	N4	-178.2(3)
C3	C4	C5	N1	-0.1(5)	C17	C16	C21	C20	0.7(4)
C3	C4	C6	O1	-63.4(4)	C17	C18	C19	C20	0.6(5)
C5	N1	C1	C2	2.0(5)	C18	C19	C20	C21	0.3(4)
C5	C4	C6	O1	116.3(3)	C19	C20	C21	N4	178.0(3)
C6	O1	C7	O2	4.1(4)	C19	C20	C21	C16	-1.0(4)
C6	O1	C7	N2	-176.2(3)	C21	N4	C22	C23	145.2(3)
C6	C4	C5	N1	-179.9(3)	C21	N4	C25	C24	-117.9(3)
C7	O1	C6	C4	168.0(3)	C21	C16	C17	C18	0.1(4)
C7	N2	C8	C9	-88.6(3)	C22	N4	C21	C16	105.7(3)
C8	N2	C7	O1	-178.0(2)	C22	N4	C21	C20	-73.2(3)
C8	N2	C7	O2	1.6(5)	C22	N4	C25	C24	7.8(3)
C8	C9	C10	C11	-175.6(3)	C22	C23	C24	C25	42.2(4)
C8	C9	C14	C13	176.3(3)	C23	C24	C25	N4	-30.7(4)
C9	C10	C11	C12	-0.4(4)	C25	N4	C21	C16	-132.2(3)
C10	C9	C14	C13	-0.6(4)	C25	N4	C21	C20	48.8(4)
C10	C11	C12	N3	-178.4(3)	C25	N4	C22	C23	18.1(3)

Table S7 Hydrogen Atom Coordinates (Å×10^4) and Isotropic Displacement Parameters (Å^2×10^3) for F8·2HCl.

Atom	*x*	*y*	*z*	U(eq)
H1	-391.42	4846.95	1592.24	64
H2	6096.04	8362.1	4799.86	39
H3	9072.28	7839.39	9708.95	34
H4	13405.74	8235.63	10493.92	38
H1A	-1472.74	5361.53	2894.18	67
H2A	-134.04	5912.01	4546.7	64
H3A	2263.25	5820.44	4805.97	55
H5	1882.01	4695.79	1743.85	54
H6A	4073.9	4580.53	3882.11	50
H6B	4117.98	4839.61	2769.21	50
H8A	8450.87	9116.36	5175.29	41
H8B	8672.31	7685.13	4793.28	41
H10	6623.3	8192.03	6703.69	36
H11	7332.21	8230.08	8430.87	33
H13	11316.1	8075.86	7992.08	34
H14	10600.89	8072.12	6279.01	35

Table S7 Hydrogen Atom Coordinates (Å×10[4]) and Isotropic Displacement Parameters (Å[2]×10[3]) for F8·2HCl.

Atom	*x*	*y*	*z*	U(eq)
H17	9138.03	8623.18	11272.82	38
H19	11566.71	8482.9	13926.51	39
H20	13500.4	8355.14	13108.15	38
H22A	14687.94	10203.25	11116.81	53
H22B	14729.82	10149.06	12292.47	53
H23A	16473.81	9080.08	10972.1	59
H23B	16992.91	9935.13	12137.41	59
H24A	16709.22	7544.37	11897.36	54
H24B	16014.59	8270.74	12790.02	54
H25A	14056.1	6829.65	11852.15	47
H25B	14599.14	6787.45	10794.61	47
