# Supplementary material for: Development of o-aminobenzamide salt derivatives for improving water solubility and anti-undifferentiated gastric cancer
Source: Front Pharmacol. 2023 Jul 11;14:1118397. doi: 10.3389/fphar.2023.1118397 (PMC10368370; doi:10.3389/fphar.2023.1118397)

Supplementary Material File 1: NMR Data for Compound 3-6, F8 and F8·2HCl

The proton chemical shifts (δ) were reported relative to the residual solvent peak, TMS was used as an internal standard (DMSO-D6 at 2.50 ppm), and the chemical shifts of the carbon spectrum were also reported relative to the residual solvent peak (DMSO-d_6 at 39.5 ppm). The following abbreviations are used to indicate diversity: s = singlet, d = doublet, t = triplet, q = quartet, quint = quintet, m = multiple, br = broad.

Compound 3: methyl-5-fluoro-2-(pyrrolidin-1-yl)benzoate

^{1}H NMR (500 MHz, $CDCl_3$, ppm) δ 7.30 (d, J = 9.1 Hz, 1H), 7.05 (t, J = 8.5 Hz, 1H), 6.73 (dd, J = 9.3, 4.5 Hz, 1H), 3.89 (s, 3H), 3.19 (t, J = 6.3 Hz, 4H), 1.94 (t, J = 6.3 Hz, 4H). ^{13}C NMR (126 MHz, $CDCl_3$, ppm) δ 168.4 (d, J = 2.3 Hz), 154.1 (d, J = 235.4 Hz), 145.2, 119.1 (d, J = 22.4 Hz), 117.3 (d, J = 6.2 Hz), 117.1 (d, J = 23.8 Hz), 115.1 (d, J = 7.2 Hz)., 52.3, 51.3, 26.0.

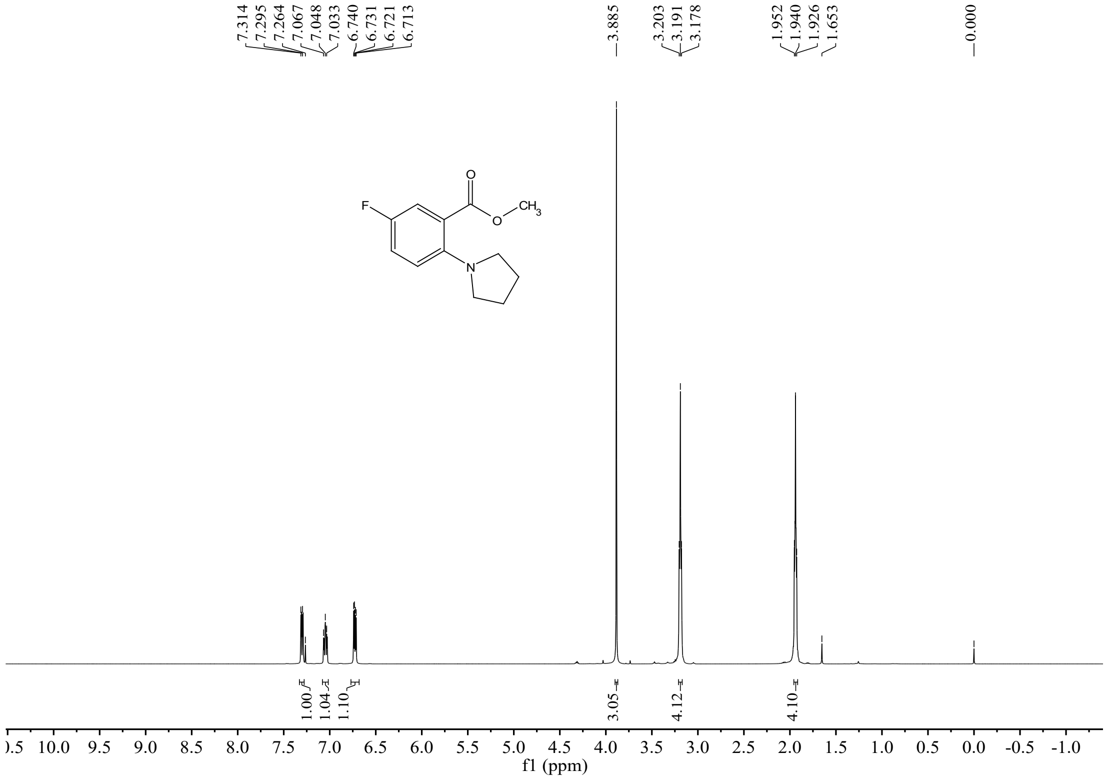

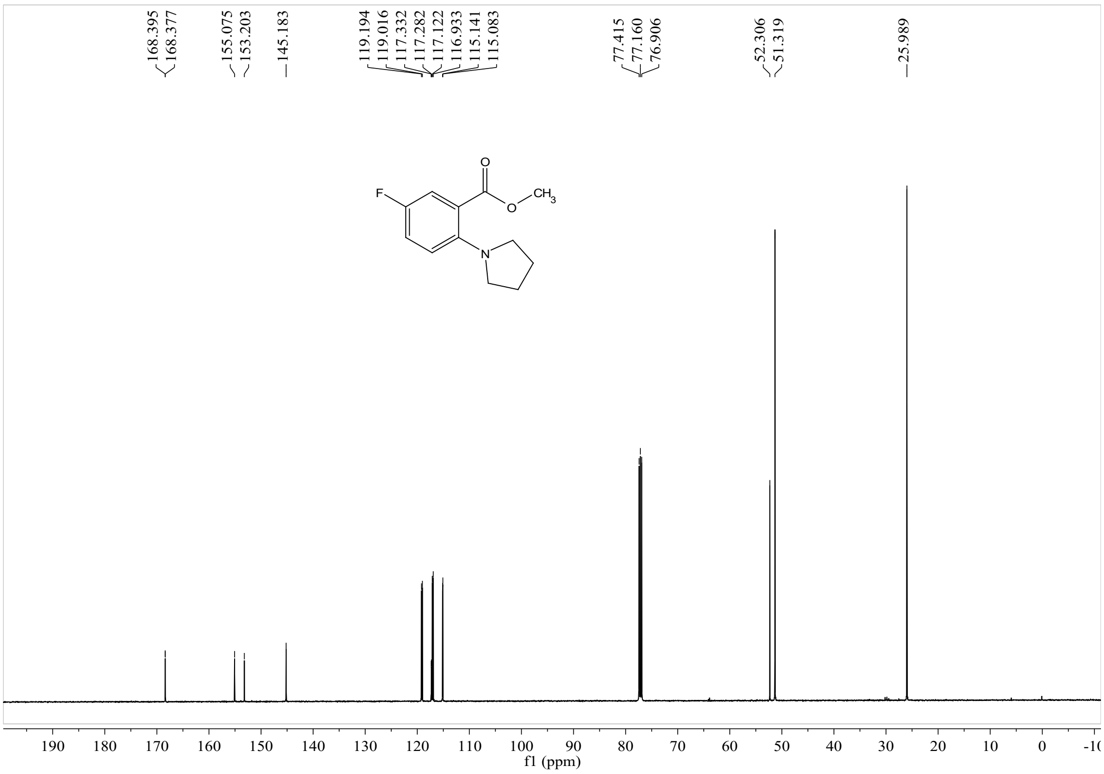

Compound 4: 5-fluoro-2-(pyrrolidin-1-yl)benzoic acid

^{1}H NMR (500 MHz, $CDCl_3$, ppm) δ 7.96 (d, J = 8.8 Hz, 1H), 7.51 (dd, J = 8.9, 4.5 Hz, 1H), 7.34 – 7.26 (m, 1H), 3.29 (t, J = 6.4 Hz, 4H), 2.18 (t, J = 3.9 Hz, 4H).

^{13}C NMR (126 MHz, $CDCl_3$, ppm) δ 166.8 (d, J = 2.1 Hz), 161.26 (d, J = 249.4 Hz), 143.7 (d, J = 3.2 Hz), 128.8 (d, J = 7.4 Hz), 124.8 (d, J = 8.2 Hz), 121.2 (d, J = 23.3 Hz), 118.2 (d, J = 23.9 Hz), 56.1, 24.9.

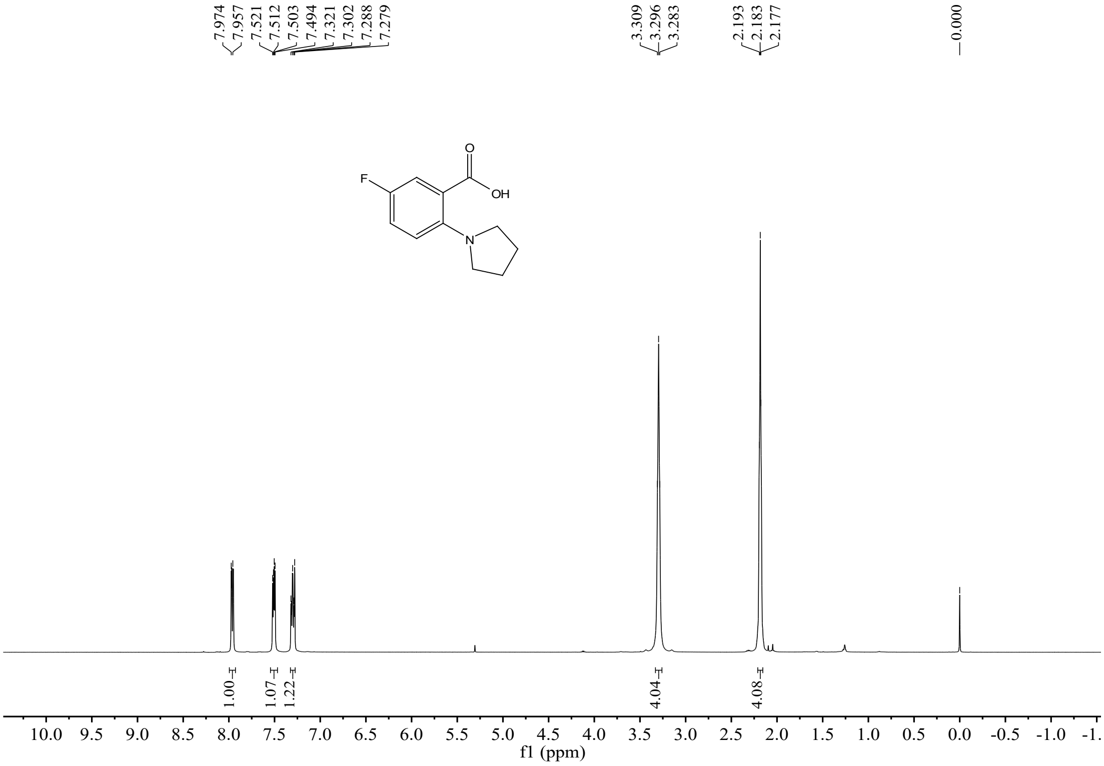

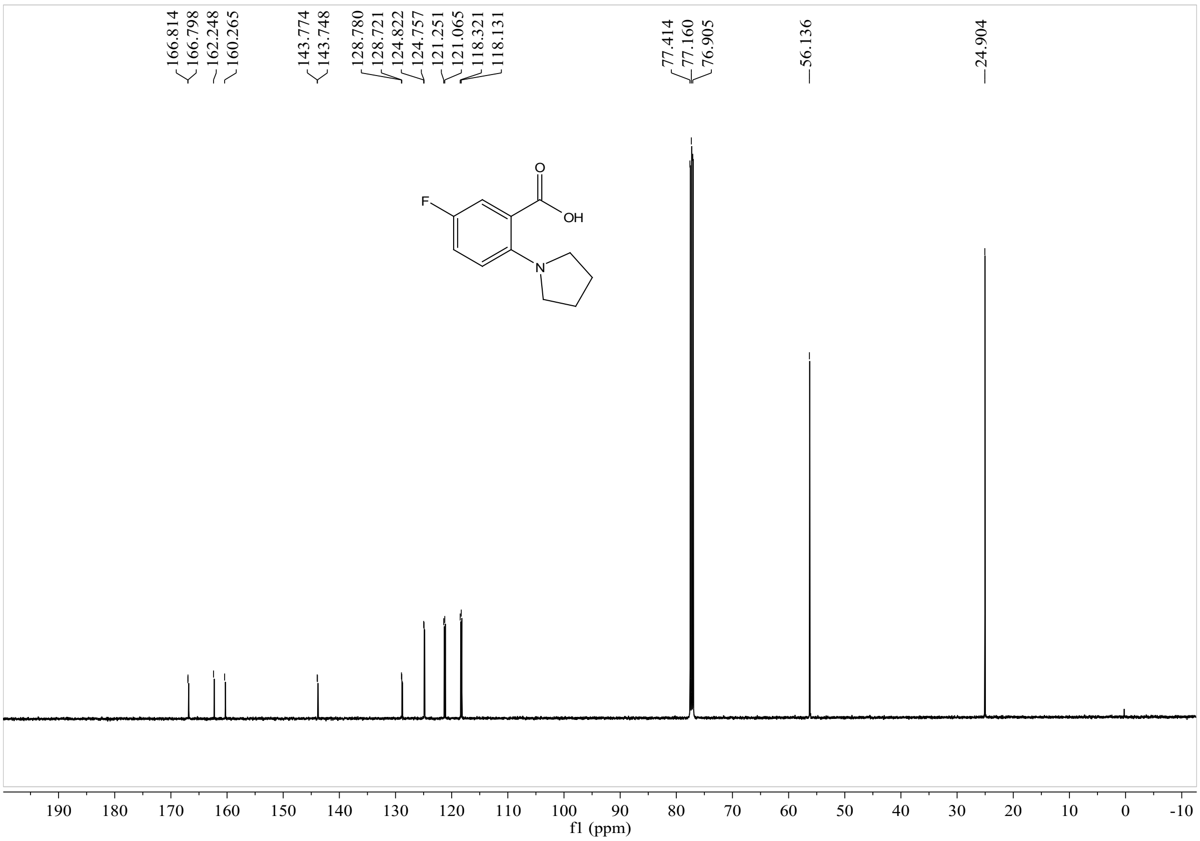

Compound 6: pyridin-3-ylmethyl (4-aminobenzyl)carbamate

^{1}H NMR (500 MHz, $CDCl_3$, ppm) δ 8.57 (s, 1H), 8.53 (d, J = 4.9 Hz, 1H), 7.67 (d, J = 7.9 Hz, 1H), 7.28 – 7.25 (m, 1H), 7.06 (d, J = 7.9 Hz, 2H), 6.62 (d, J = 7.9 Hz, 2H), 5.12 (s, 2H), 4.24 (d, J = 5.8 Hz, 2H). ^{13}C NMR (126 MHz, $CDCl_3$, ppm) δ 156.1, 149.5, 149.4, 146.0, 136.0, 132.4, 129.0, 128.1, 123.5, 115.3, 64.2, 44.9.

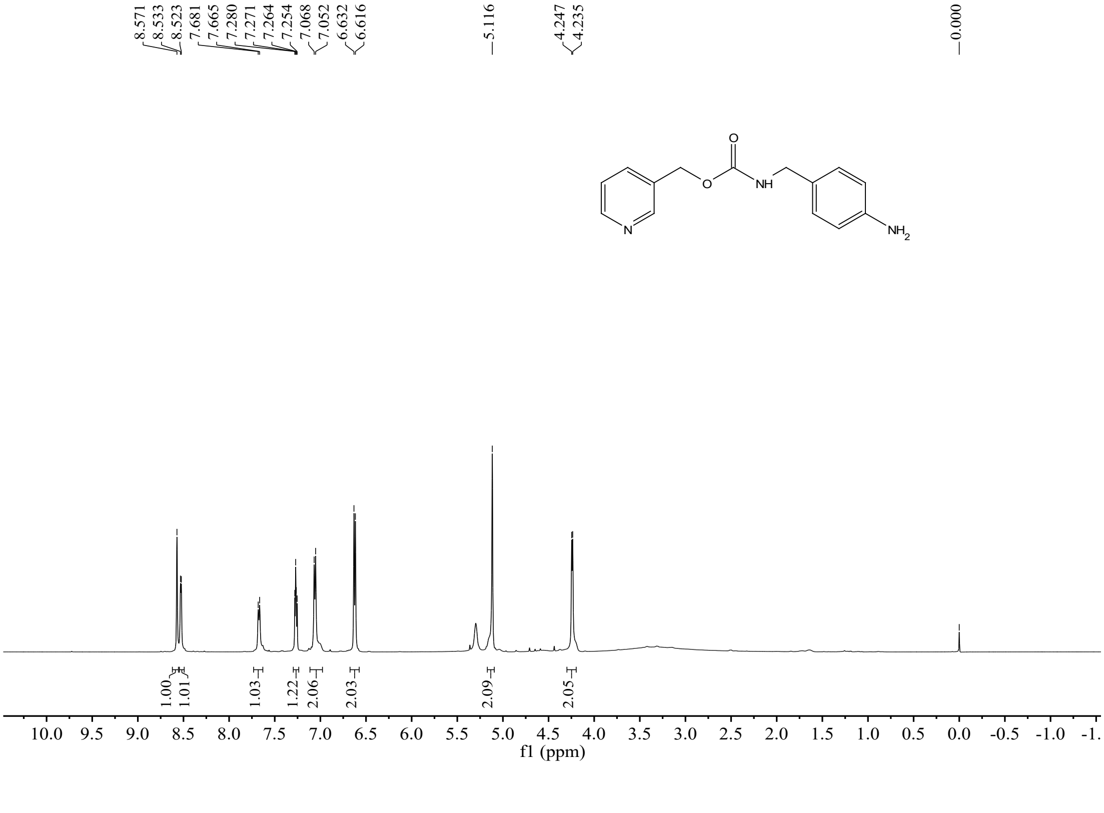

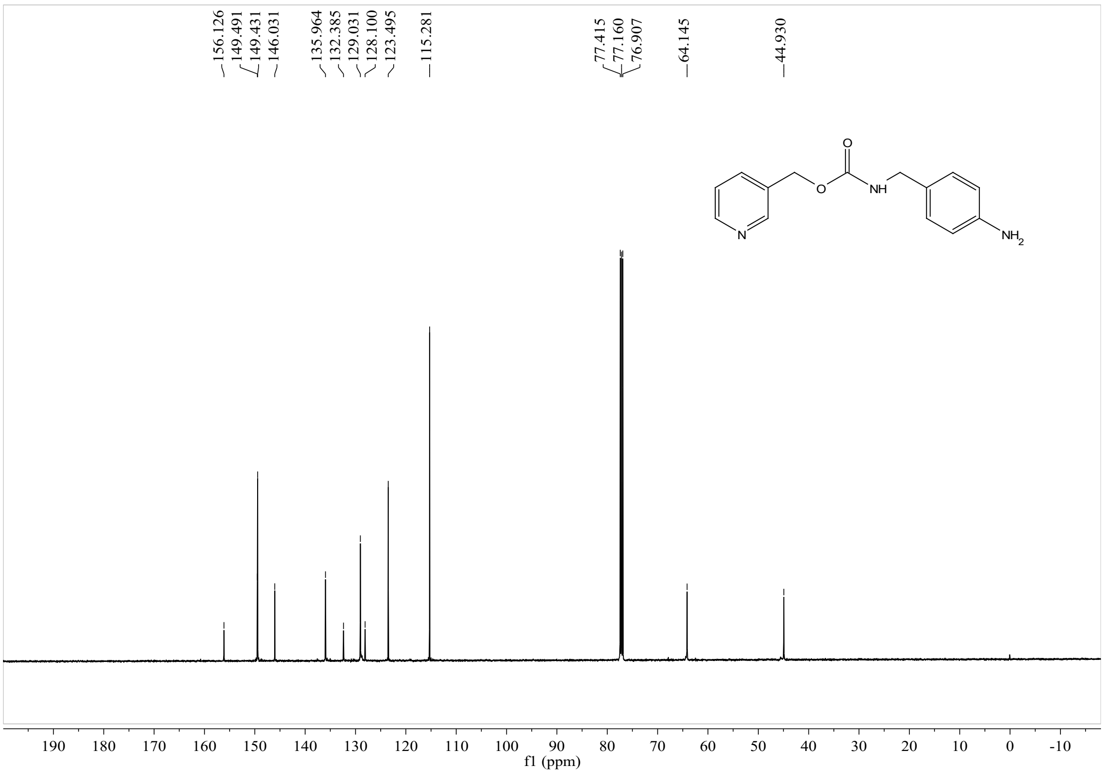

Compound F8: pyridin-3-ylmethyl (4-(5-fluoro-2-(pyrrolidin-1-yl)benzamido)benzyl) carbamate

^{1}H NMR (500 MHz, $CDCl_3$, ppm) δ 12.08 (s, 1H), 8.61 (s, 1H), 8.55 (d, *J* = 4.8 Hz, 1H), 7.89 (d, *J* = 9.6 Hz, 1H), 7.70 (d, *J* = 7.9 Hz, 1H), 7.61 (d, *J* = 8.1 Hz, 2H), 7.30 – 7.23 (m, 4H), 7.14 (t, *J* = 8.0 Hz, 1H), 5.15 (s, 2H), 4.36 (d, *J* = 6.0 Hz, 2H), 3.16 (t, *J* = 5.0 Hz, 4H), 2.06 (t, *J* = 5.0 Hz, 4H).

^{13}C NMR (125 MHz, $CDCl_3$) δ 163.5 (d, *J* = 2.0 Hz), 159.5 (d, *J* = 244.0 Hz), 156.2, 149.6, 149.6, 144.6 (d, *J* = 2.8 Hz), 138.2, 136.0, 134.0, 132.3, 130.0 (d, *J* = 6.9 Hz), 128.6, 123.5, 122.2 (d, *J* = 7.7 Hz), 120.1, 119.1 (d, *J* = 22.6 Hz), 117.8 (d, *J* = 24.3 Hz), 64.3, 53.9, 44.9, 24.7.

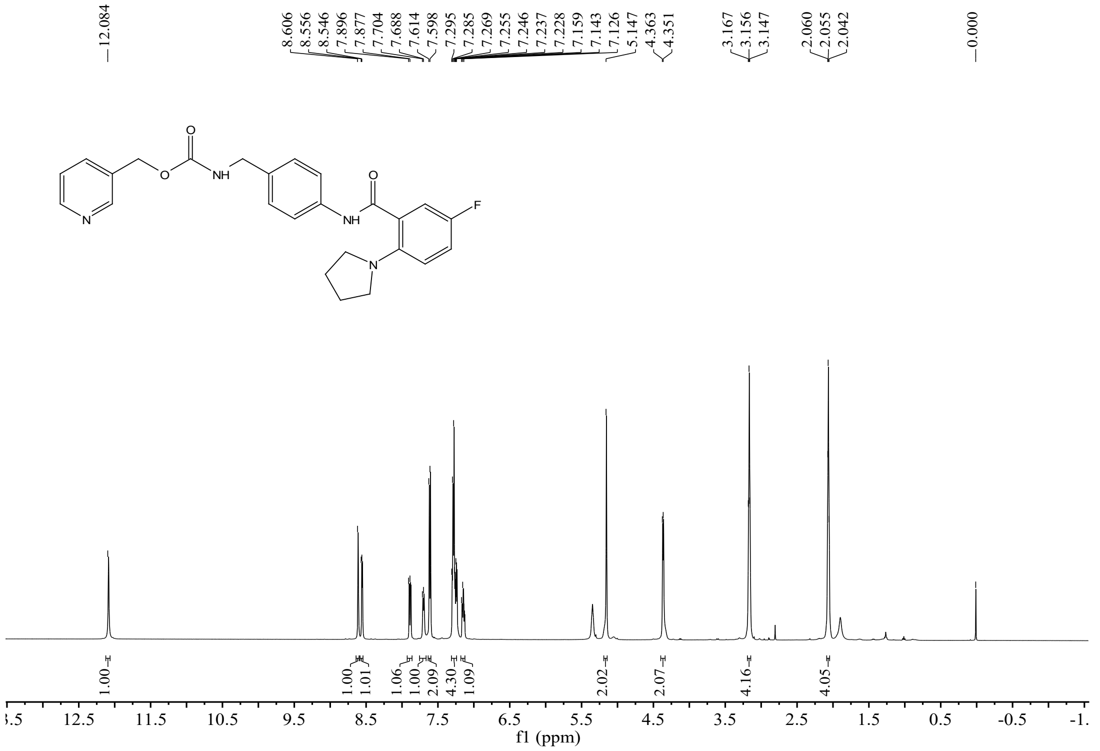

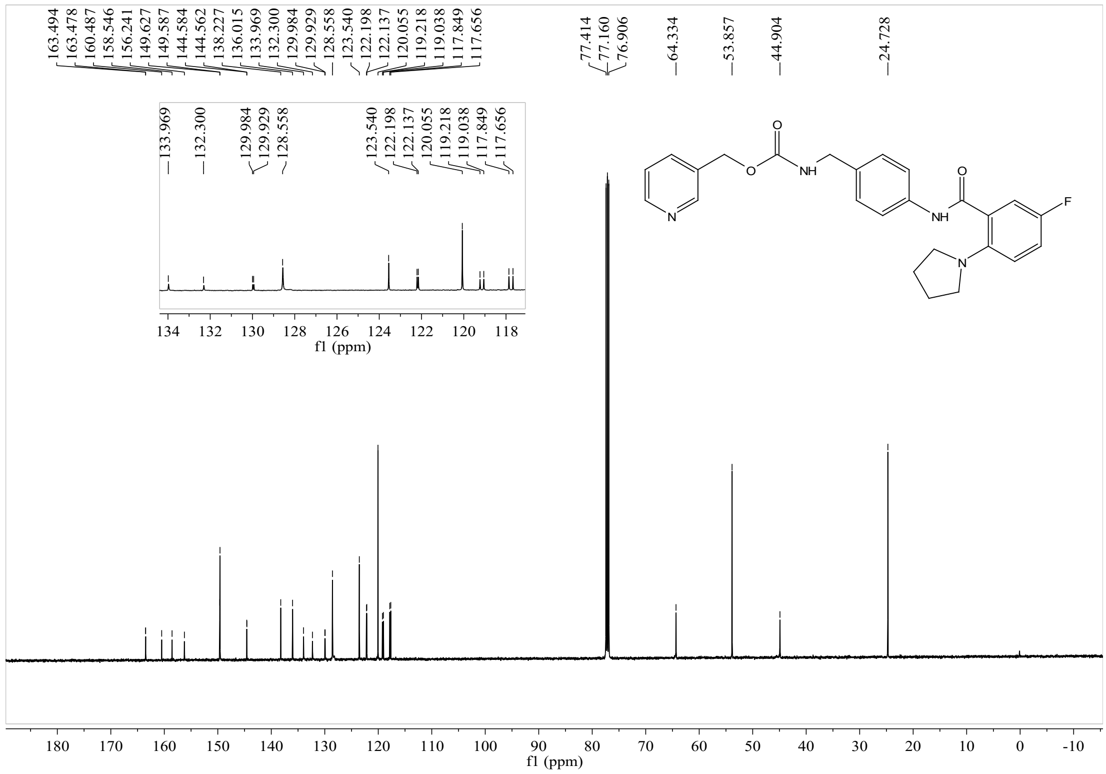

Compound F8

^{1}H NMR (500 MHz, DMSO-d_6, ppm) δ 10.51 (s, 1H), 8.59 (s, 1H), 8.53 (d, J = 4.9 Hz, 1H), 7.85 (t, J = 6.1 Hz, 1H), 7.78 (d, J = 7.9 Hz, 1H), 7.64 (d, J = 8.2 Hz, 2H), 7.40 (t, J = 6.3 Hz, 1H), 7.23 – 7.14 (m, 4H), 6.82 (dd, J = 8.9, 4.5 Hz, 1H), 5.09 (s, 2H), 4.17 (d, J = 6.1 Hz, 2H), 3.18 (t, J = 6.2 Hz, 4H), 1.85 (t, J = 6.5 Hz, 4H).

^{13}C NMR (125 MHz, DMSO-d_6, ppm) δ 166.7, 156.2, 154.0 (d, J = 234.3 Hz), 149.1 (d, J = 5.9 Hz), 143.0, 138.0, 135.8, 134.8, 132.8, 127.5, 124.5 (d, J = 5.6 Hz), 123.5, 119.5, 116.9 (d, J = 21.4 Hz), 115.8 (d, J = 7.3 Hz), 115.4 (d, J = 23.0 Hz), 63.2, 50.1, 43.5, 25.2.

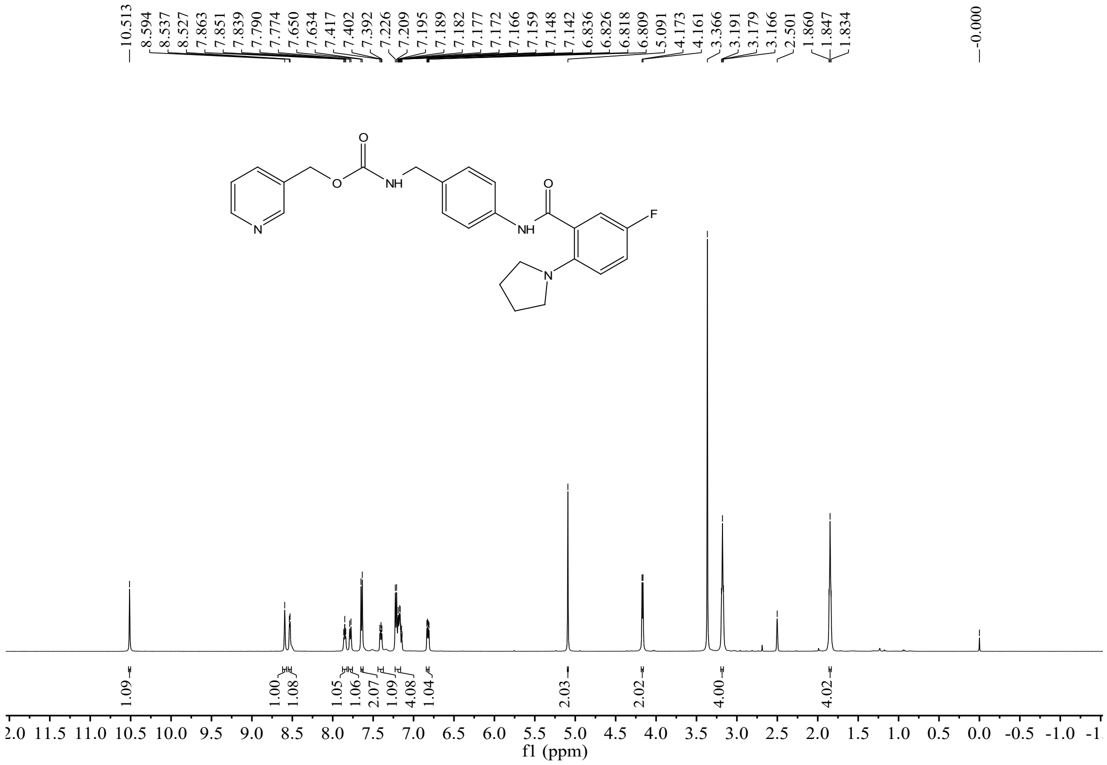

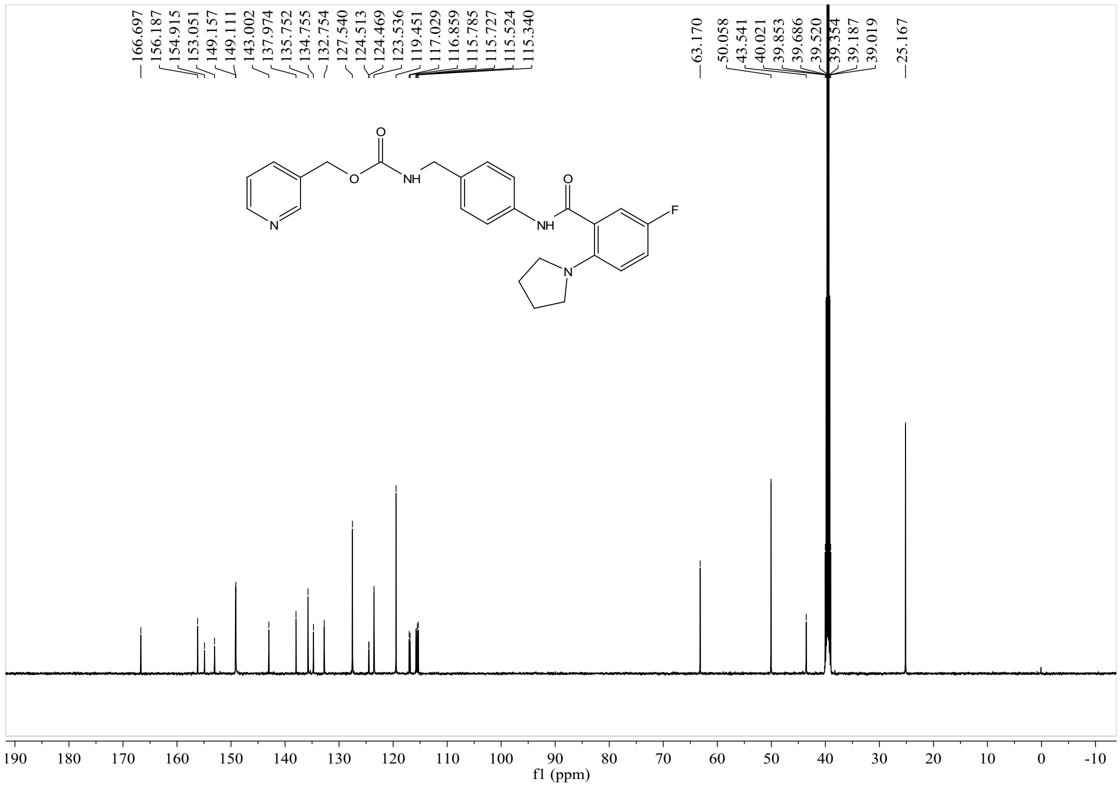

Compound F8·2HCl

^{1}H NMR (500 MHz, DMSO-d_6, ppm) δ 10.66 (s, 1H), 8.94 (s, 1H), 8.91 (d, J = 5.8 Hz, 1H), 8.58 (d, J = 8.0 Hz, 1H), 8.11 (t, J = 6.8 Hz, 1H), 8.06 (t, J = 6.2 Hz, 1H), 7.66 (d, J = 8.2 Hz, 2H), 7.33 (d, J =

8.6 Hz, 1H), 7.28 – 7.10 (m, 4H), 7.10 (s, 1H), 5.27 (s, 2H), 4.19 (d, *J* = 6.0 Hz, 2H), 3.32 (t, *J* = 6.5 Hz, 4H), 1.91 (t, *J* = 6.3 Hz, 5H).

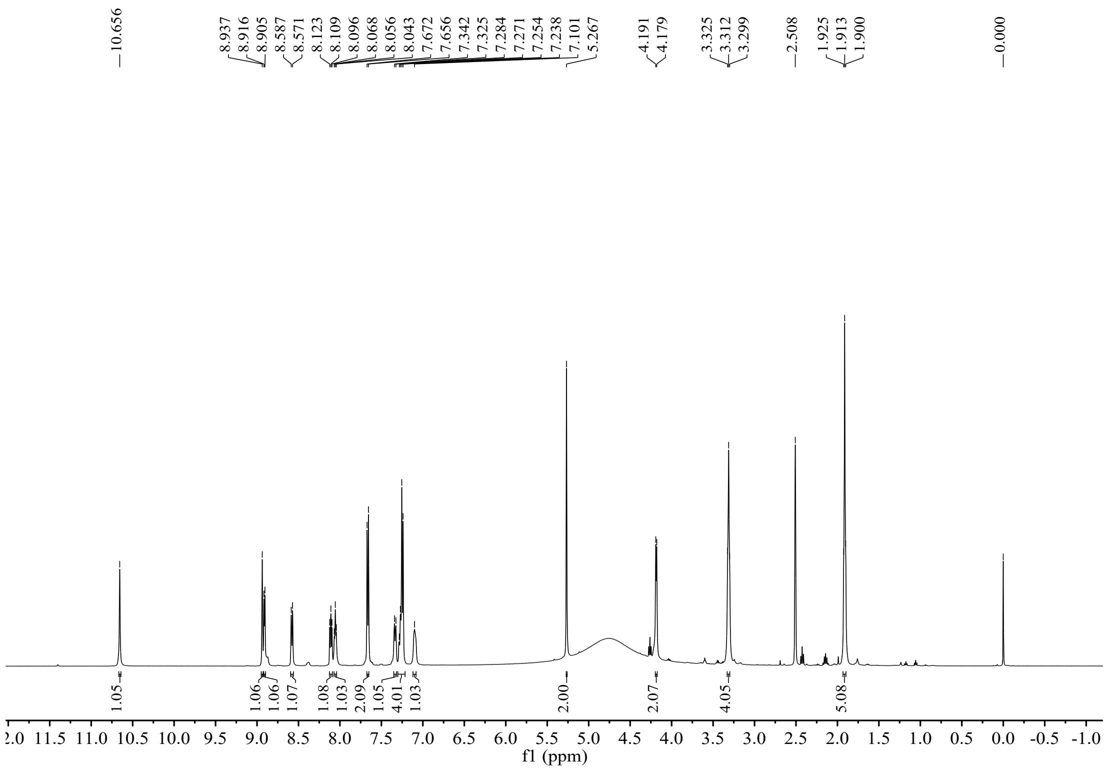

Supplement: Supplementary file 3 [file DataSheet1.pdf]
